# A Venom Allergen-Like Protein, RsVAP, the First Discovered Effector Protein of *Radopholus similis* That Inhibits Plant Defense and Facilitates Parasitism

**DOI:** 10.3390/ijms22094782

**Published:** 2021-04-30

**Authors:** Junyi Li, Chunling Xu, Sihua Yang, Chun Chen, Shiqiao Tang, Jiafeng Wang, Hui Xie

**Affiliations:** Research Center of Nematodes of Plant Quarantine, Laboratory of Plant Nematology, Department of Plant Pathology/Guangdong Province Key Laboratory of Microbial Signals and Disease Control, College of Plant Protection, South China Agricultural University, Guangzhou 510642, China; leizeonngai1206@163.com (J.L.); xuchunling@scau.edu.cn (C.X.); evayeung0605@163.com (S.Y.); chchun@scau.edu.cn (C.C.); tangshiqiao666@163.com (S.T.); jfwang@scau.edu.cn (J.W.)

**Keywords:** *Radopholus similis*, venom allergen-like protein, plant defense, interacting protein

## Abstract

*Radopholus similis* is a migratory endoparasitic nematode that is extremely harmful to host plants. Venom allergen-like proteins (VAPs) are members of the cysteine-rich secretory protein family that are widely present in plants and animals. In this study, we cloned a VAP gene from *R. similis*, designated as RsVAP. RsVAP contains an open reading frame of 1089 bp encoding 362 amino acids. RsVAP is specifically expressed in the esophageal gland, and the expression levels of RsVAP are significantly higher in juveniles than in other life stages of *R. similis*. This expression pattern of RsVAP was consistent with the biological characteristics of juveniles of *R. similis*, which have the ability of infection and are the main infection stages of *R. similis*. The pathogenicity and reproduction rate of *R. similis* in tomato was significantly attenuated after RsVAP was silenced. In tobacco leaves transiently expressing RsVAP, the pathogen-associated molecular pattern-triggered immunity (PTI) induced by a bacterial flagellin fragment (flg22) was inhibited, while the cell death induced by two sets of immune elicitors (BAX and Gpa2/RBP-1) was repressed. The RsVAP-interacting, ras-related protein RABA1d (LeRabA1d) was identified in tomato hosts by yeast two-hybrid and co-immunoprecipitation assays. RsVAP may interact with LeRabA1d to affect the host defense response, which in turn facilitates nematode infection. This study provides the first evidence for the inhibition of plant defense response by a VAP from migratory plant-parasitic nematodes, and, for the first time, the target protein of *R. similis* in its host was identified.

## 1. Introduction

*Radopholus similis* (Cobb, 1893) Thorne, 1949 is one of the world’s ten most important plant-parasitic nematodes [1]. As a migratory endoparasitic nematode, *R. similis* is extremely harmful to its host plants. All life stages of *R. similis* occur inside the roots. *R. similis* use their stylets to enter the host and migrate through host tissues. During this process, the nematode movement and secretions of stylets destroy host cells and tissues, causing extensive damage, including dark lesions, tissue necrosis, slow-growing, plant wilting and plant lodging [1]. This nematode is, therefore, listed as a quarantine pest in many countries [2]. *R. similis* can parasitize more than 250 plant species, including banana (*Musa* spp.), citrus (*Citrus* spp.), pepper (*Piper nigrum*), coconut (*Cocos nucifera*), vegetables, ornamental plants and many other crops that are important in global commerce [3]. Because of its broad host range, *R. similis* causes huge losses in global agricultural production.

When infecting plants, plant-parasitic nematodes can secrete proteins or molecules through their stylet or body wall into plant cells. Most of these secretions are proteins produced by their esophageal glands, while some secretory proteins come from the head sensilla and tail [4,5]. These secretory proteins are essential for the successful host infection by nematodes, and genes encoding these proteins are known as effector protein genes or effectors [6]. More than 100 plant nematode effector protein genes have been cloned and studied [7]. In general, the functions of plant nematode effectors include the degradation and modification of plant cell walls, the inhibition of host defense response and regulating host signaling pathways [5]. Some studies have reported on the effectors of *R. similis*, whose genes play a crucial role in host infection and pathogenesis [8,9,10,11,12,13,14,15].

Venom allergen-like proteins (VAPs) that exist widely in animals and plants are members of the cysteine-rich secretory protein (CAP) family, characterized by highly conserved cysteine residues at the carboxyl terminus. Hawdon et al. [16] cloned the first animal-derived *VAP* gene, *AcASP1*, from the animal-parasitic nematode *Ancylostoma caninum*. *AcASP1* encoded protein was found to be secreted into the host only by infective third-stage larvae, indicating that *AcASP1* plays an essential role in host infection by the nematode. Subsequently, this gene was also discovered in another animal-parasitic nematode, *Hemonchus contortus*, and its encoded protein was localized in the nematode pharyngeal cavity; thus, the protein may be secreted into the host to exert its function [17].

The first *VAP* gene discovered in plant-parasitic nematodes was *Mimsp1* in *Meloidogyne incognita*. *Mimsp1* is expressed in second-stage juvenile both before and after infection but not in females, suggesting that this gene plays a role in the infective stage of the nematode [18]. In addition to *M. incognita*, *VAP* genes have been identified in other plant-parasitic nematodes, such as *Bursaphelenchus xylophilus* [19,20,21], *B. mucronatu* [21], *Ditylenchus africanus* [22], *D. destructor* [23], *Globodera rostochiensis* [24,25], *Heterodera schachtii* [25], *H. glycines* [26] and *M. hispanica* [27]. All of the known *VAP* genes from these plant-parasitic nematodes encode signal peptide-containing proteins and are specifically expressed in the esophageal glands, which may be related to nematode parasitism.

*MhiVAP1* of *M. hispanica* is expressed in the subventral esophageal glands of second-stage juvenile, and tomato root exudates can induce high expression in second-stage juvenile before infection [27,28]. After silencing *MhiVAP1* using a double-stranded RNA (dsRNA) solution, the pathogenicity of *M. hispanica* on plants was markedly reduced [29]. Based on expression analysis of three *VAP* genes in different life stages of *B. xylophilus*, *BxVAP1* expression levels were the highest in nematodes migrating and reproducing on pine trees. The migration rate of *B. xylophilus* was considerably reduced after silencing *BxVAP1*, suggesting that *BxVAP1* expression is conducive to nematode parasitism in an early stage [19,20]. Hence, far, many studies exploring the function of *VAP* genes from plant-parasitic nematodes have mainly focused on sedentary root-knot nematodes and cyst nematodes, whereas migratory nematodes have been less studied. In particular, there are no reports on *VAP* genes from *R. similis*.

In the present study, a novel *VAP* gene, designated *RsVAP*, was cloned from *R. similis*. To clarify the spatiotemporal expression patterns of *RsVAP* in *R. similis* and its role in host pathogenesis, we performed developmental expression pattern analysis, in situ hybridization, Southern blot, and RNA interference (RNAi) assays. We also determined the inhibitory effects of RsVAP on pathogen-associated molecular pattern-triggered immunity (PTI) induced by bacterial flagellin (flg22) and the cell death induced by two sets of immune elicitors (BAX and Gpa2/RBP-1) to clarify whether *RsVAP* can inhibit plant defense response. The targets of RsVAP in host tomatoes were screened using the yeast two-hybrid (Y2H) assay. This study presents the first evidence for the inhibitory effect of *R. similis* on plant defense responses and identifies the first host plant proteins interacting with *R. similis*.

## 2. Results

### 2.1. Cloning and Sequence Characteristics of the Full-Length RsVAP Gene

**Figure 1:**
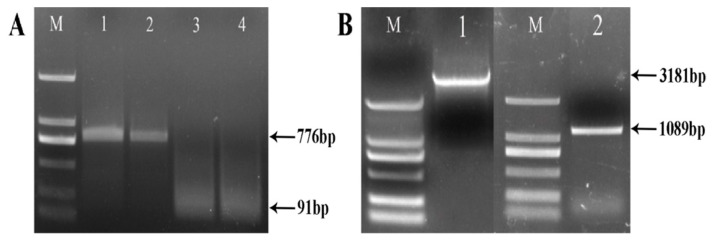
PCR amplification of *RsVAP* of *Radopholus similis*. (**A**) RACE amplification of *RsVAP*, M: DS2000 marker; 1–2: 5′ end of *RsVAP*; 3–4: 3′ end of *RsVAP*. (**B**) Full-length amplification of *RsVAP*, M: DS2000 marker; 1: full-length DNA of *RsVAP*; 2: full-length cDNA of *RsVAP.*

Based on the transcript sequence of a suspected *VAP* gene in the *R. similis* transcriptome, we performed 5′- and 3′-RACE using the designed primers, with 5′- and 3-RACE-ready cDNA as templates, respectively. A 776-bp fragment was amplified from the 5′ end of *RsVAP*, whereas a 91-bp fragment containing a poly(A) tail was amplified from the 3′ end of the gene (Figure 1). The amplified products were sequenced and spliced into known transcript sequences, yielding a 1300 bp full-length cDNA sequence of *RsVAP*. The full-length cDNA of *RsVAP* was predicted to contain an ORF of 1089 bp encoding a protein of 362 aa.

**Figure 2:**
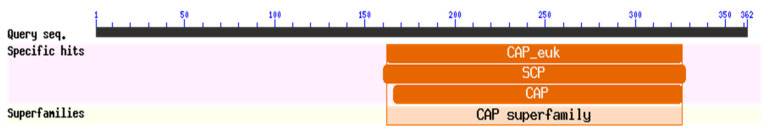
Prediction of conserved domains of RsVAP of *Radopholus similis.*

Based on the predicted ORF sequence of *RsVAP*, we designed primers for the full-length amplification of *RsVAP*, using the cDNA and DNA of *R. similis* as templates. After sequencing, the full-length DNA of *RsVAP* was 3181 bp, and it contained an ORF of 1089 bp (Figure 1). The DNA sequence comprised four intron regions. Through the BLASTx searches in NCBI, we found that the aa sequence of RsVAP contained a conserved domain of the cysteine-rich secretory protein (CAP) family (Figure 2). The aa sequence of the RsVAP also exhibited the highest sequence similarity (42%) with the aa sequence of an SCP-like protein in the animal-parasitic nematode *Oesophagostomum dentatum*. The RsVAP also showed aa sequence similarities to VAPs from other plant-parasitic nematodes, including DdVAP1 and DdVAP2 of *D. destructor* (38.00% and 37.32%, respectively), HaVAP1 of *H. avenae* (37.74%), and GrVAP1 of *G. rostochiensis* (37.26%). Moreover, the RsVAP was predicted to contain a 25-aa signal peptide with no transmembrane domain (Supplementary Figure S1). These results suggest that the RsVAP may be secreted and exert its functions in the host tissue.

**Figure 3:**
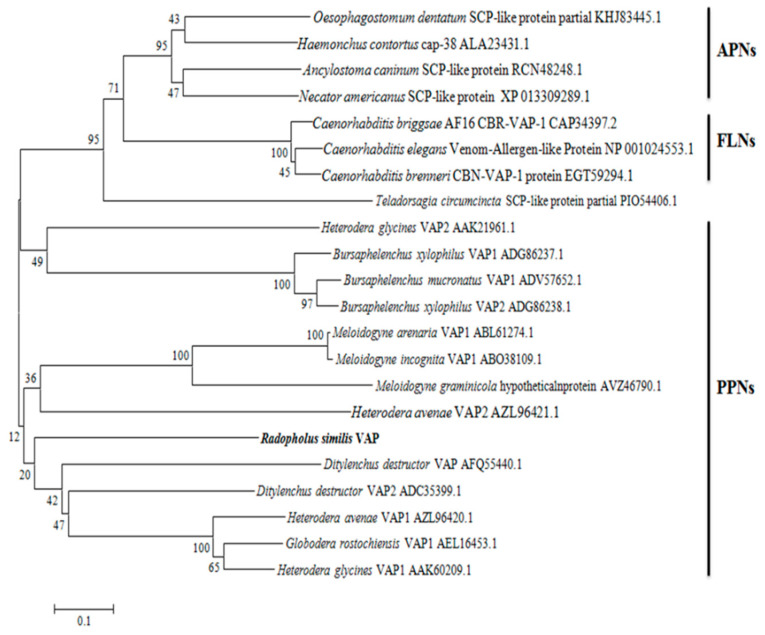
Neighbor-joining tree of RsVAP from *Radopholus similis* and related proteins from other nematode species. The phylogenetic tree for proteins with conserved domains of cysteine-rich secretory proteins from free-living nematodes, animal-parasitic nematodes, and plant-parasitic nematodes was generated using MEGA 6.0. The deduced amino acid sequence of *RsVAP* is marked in bold, and each sequence of other nematode species is followed by its accession number in GenBank. The bar indicates a 10% sequence variance.

We then conducted a phylogenetic analysis based on the aa sequence of the RsVAP obtained in this study and 21 VAP aa sequences of 17 other nematode species retrieved from the NCBI database. The VAPs of these nematodes formed three clusters in the neighbor-joining tree (Figure 3). The RsVAP was clustered with the VAPs of other plant-parasitic nematodes, whereas the VAPs of animal-parasitic nematodes and those of free-living nematodes clustered separately. These results suggest that VAPs may have specific functions related to the life habits of nematodes, and the VAPs of different nematodes in the same living habitats may have evolved in the same direction so that nematodes could better adapt to their living habitats.

### 2.2. Developmental Expression Patterns of RsVAP in R. similis

**Figure 4:**
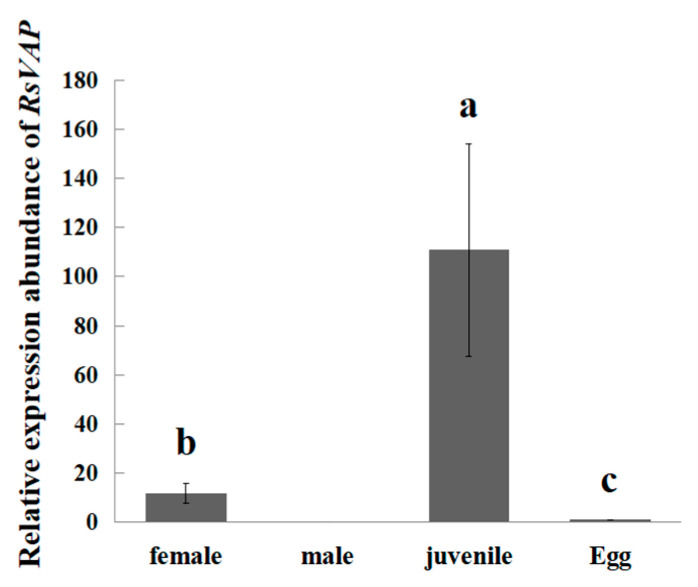
Relative expression levels of *RsVAP* in different life stages of *Radopholus similis.* The *x*-axis represents the life stage of *R. similis*, and the *y*-axis represents the relative expression level of *RsVAP*. Bars indicate standard errors of the mean (*n* = 3), and a–c above the bars indicate significant differences (*p* < 0.05) among groups.

Using RT–qPCR, we analyzed the relative expression levels of *RsVAP* in different life stages of *R. similis*. *RsVAP* expression was detected in females, juveniles, and eggs, but not in males (Figure 4). The expression levels of *RsVAP* in juveniles were significantly higher than those in the other life stages (*p* < 0.05), i.e., 9.56- and 108.02-fold compared with females and eggs, respectively. The expression levels of *RsVAP* were also significantly higher (11.30-fold) in females than in eggs (*p* < 0.05).

### 2.3. Tissue Localization of RsVAP in R. similis

**Figure 5:**
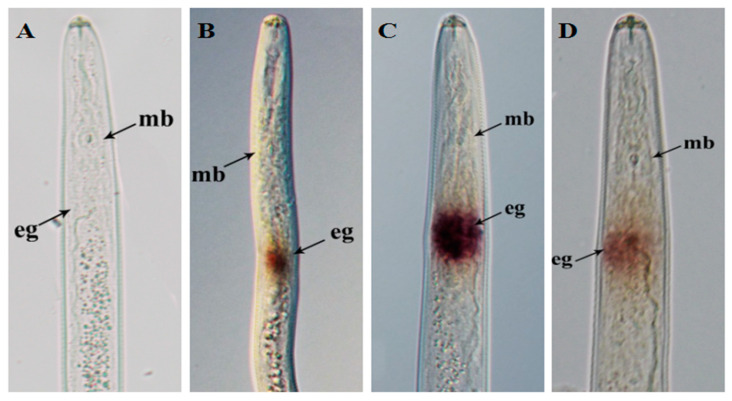
Tissue localization of RsVAP in *Radopholus similis* via in situ hybridization. (**A**) No hybridization signals were detected in negative control nematodes with digoxigenin-labeled sense *RsVAP* RNA probe; (**B**–**D**) specific hybridization signals were detected in the esophageal glands of *R. similis* with digoxigenin-labeled antisense *RsVAP* RNA probe; mb: medium bulb; eg: esophageal gland.

In situ hybridization results showed that there were no specific signals in the nematodes hybridized with the sense probe (Figure 5A). However, specific hybridization signals were detected in the esophageal glands of nematodes hybridized with the antisense probe (Figure 5B–D). These results indicate that the RsVAP is specifically expressed in the esophageal glands of *R. similis*.

### 2.4. Copy Numbers of RsVAP in the R. similis Genome

**Figure 6:**
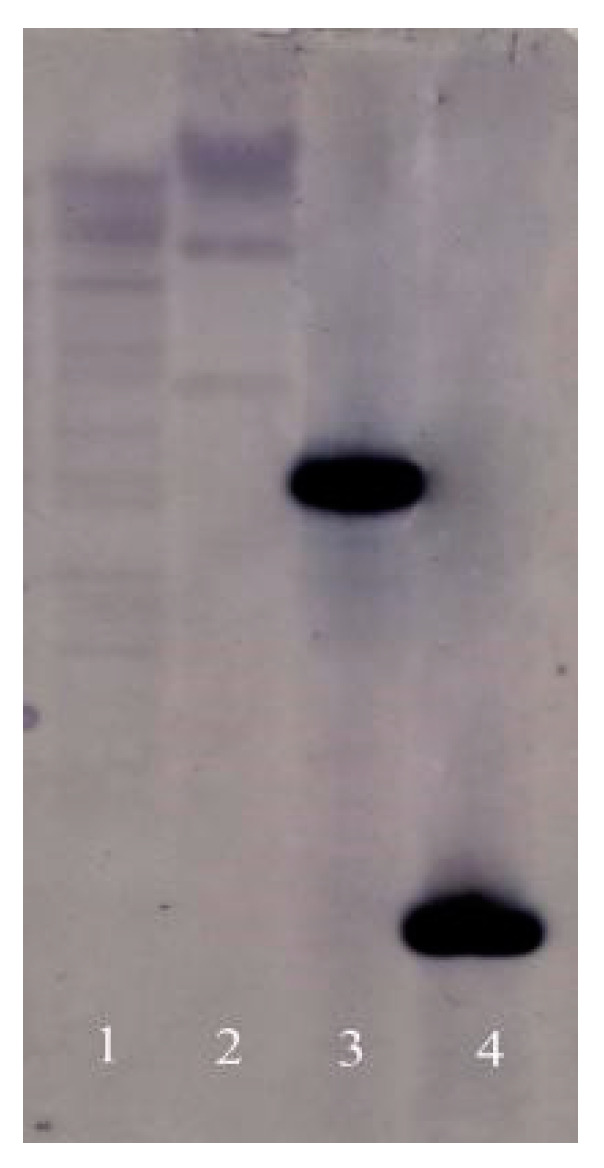
Southern blot analysis of *RsVAP* of *Radopholus similis.* 1–2: *R. similis* genome DNA digested with SacI and NcoI, respectively; 3–4: pEAST-*RsVAP* plasmid DNA digested with SacI and NcoI, respectively.

The results of the Southern blot analysis showed that, after hybridization with the probe, the genomic DNA of *R. similis* single-digested with SacI and NcoI yielded at least three hybridization bands. In contrast, only one hybridization band was present for the plasmid DNA single-digested with the same restriction enzymes (Figure 6). These results indicate that *RsVAP* exists as multiple copies in the *R. similis* genome.

### 2.5. RsVAP Inhibits Basic Defense Response in Tobacco

#### 2.5.1. RsVAP Inhibits flg22-Induced Callose Deposition

**Figure 7:**
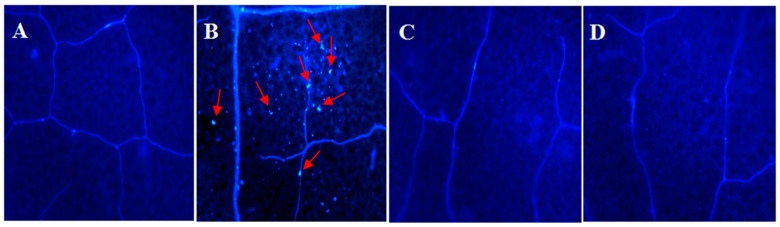
Inhibition of flg22-induced callose deposition in tobacco by RsVAP of *Radopholus similis*. (**A**) Blank control; (**B**) tobacco leaves were soaked in 10 μM flg22 solution 48 h after egfp injection; (**C**) tobacco leaves were soaked in 10 μM flg22 solution 48 h after RsVAP injection; (**D**) tobacco leaves were soaked in 10 μM flg22 solution 48 h after RsVAP^ΔSP^ injection. Calloses are marked with red arrows.

We first analyzed callose deposition in tobacco leaves injected with the cultures of *A. tumefaciens* containing the target vectors pCAMBIA1300:FLAG:Rs*VAP* and pCAMBIA1300:FLAG:Rs*VAP*^ΔSP^ (Figure 7). Compared with the egfp+flg22 treatment (Figure 7B), the number of callose deposits was significantly reduced in tobacco leaves of the RsVAP+flg22 (Figure 7C) and RsVAP^ΔSP^+flg22 (Figure 7D) treatments. These results indicate that RsVAP can inhibit flg22-induced callose deposition in tobacco leaves, regardless of the presence or absence of a signal peptide.

#### 2.5.2. RsVAP Inhibits flg22-Induced Defense Gene Expression

**Figure 8:**
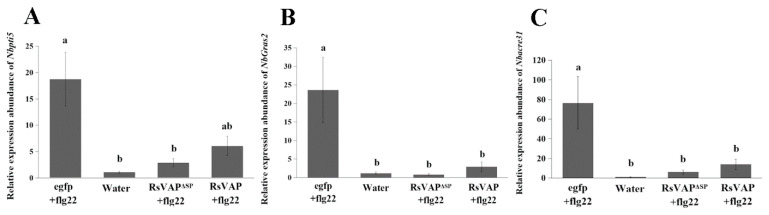
Inhibition of flg22-induced defense gene expression in tobacco by RsVAP of *Radopholus similis.* (**A**) Relative expression level of *NbPti5*; (**B**) relative expression level of *NbGras2*; (**C**) relative expression level of *Nbacre31*. The *x*-axis represents the different experimental treatments, and the *y*-axis represents the relative expression of each gene. Bars indicate standard errors of the mean (*n* = 3), and a, b above the bars indicate significant differences (*p* < 0.05) among groups.

*NbPti5*, *NbGras2* and *Nbacre31* have been demonstrated to be PTI markers in *N. benthamiana* [30]. We used RT–qPCR to analyze the relative expression levels of these three genes in tobacco leaves that had been soaked in flg22 solution after injection with *A. tumefaciens* containing the recombinant vectors. Compared with the egfp+flg22 treatment, flg22-induced upregulation of defense gene expression was inhibited in tobacco leaves of both the RsVAP+flg22 and RsVAP^ΔSP^+flg22 treatments, albeit to different extents (Figure 8).

Significant differences were detected in *NbPti5* gene expression levels between the RsVAP^ΔSP^+flg22 and egfp+flg22 treatments (*p* < 0.05), but neither between the RsVAP+flg22 and egfp+flg22 treatments (*p* > 0.05), nor between the RsVAP+flg22 and RsVAP^ΔSP^+flg22 treatments (*p* > 0.05). Compared with the water treatment, the relative gene expression levels of *NbPti5* in the RsVAP+flg22 and RsVAP^ΔSP^+flg22 treatments were both upregulated with no significant difference (*p* > 0.05).

There were significant differences in *NbGras2* expression levels between the RsVAP+flg22 or RsVAP^ΔSP^+flg22 and egfp+flg22 treatments (*p* < 0.05), but not between the former two treatments (*p* > 0.05). Compared with the water treatment, no significant difference was observed in the relative gene expression levels of *NbGras2* in the RsVAP+flg22 and RsVAP^ΔSP^+flg22 treatments (*p* > 0.05).

There were significant differences in *Nbacre31* expression levels between the RsVAP+flg22 or RsVAP^ΔSP^+flg22 and the egfp+flg22 treatment (*p* < 0.05), but not between the former two treatments (*p* > 0.05). Compared with the water treatment, the relative gene expression levels of *Nbacre31* in the RsVAP+flg22 and RsVAP^ΔSP^+flg22 treatments were both upregulated with no significant difference (*p* > 0.05).

### 2.6. RsVAP Represses BAX- and Gpa2/RBP-1-Induced Cell Death

**Figure 9:**
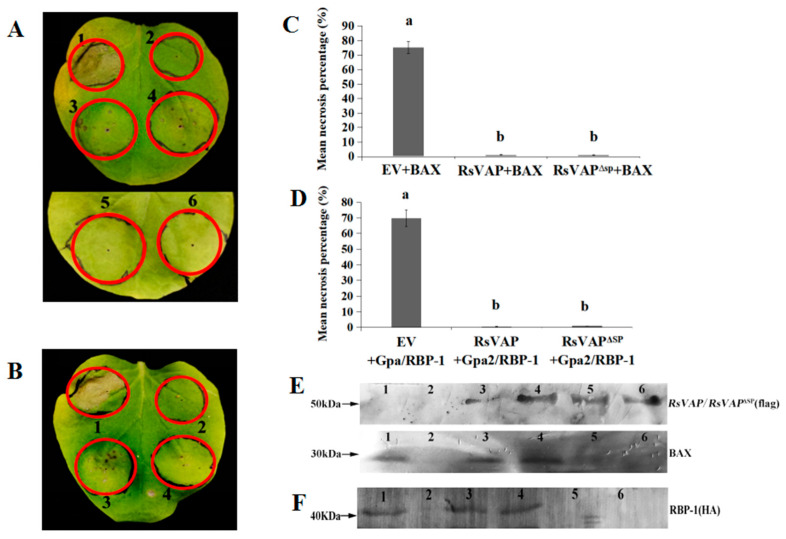
Repression of BAX/Gpa2/RBP-1-induced cell death in tobacco by RsVAP of *Radopholus similis.* (**A**) Repressive effect of RsVAP on BAX-induced cell death; (**B**) repressive effect of RsVAP on Gpa2/RBP-1-induced cell death; (**C**,**D**) mean percentage of necrosis lesions in different treatments, and a, b above the bars indicating significant differences (*p* < 0.05) among groups; (**E**,**F**) detection of protein expression by Western blot analysis; diaminobenzidine (DAB) was used for protein staining. (**A**,**E**) 1: pCAMBIA1300→24 h→BAX; 2: pCAMBIA1300; 3: RsVAP→24 h→BAX; 4: RsVAP^ΔSP^→24 h→BAX; 5: RsVAP; 6: RsVAP^ΔSP^. (**B**,**F**) 1: pCAMBIA1300→24 h→Gpa2/RBP-1; 2: pCAMBIA1300; 3: RsVAP→24 h→ Gpa2/RBP-1; 4: RsVAP^ΔSP^→24 h→Gpa2/RBP-1; 5: RsVAP; 6: RsVAP^ΔSP^.

Twenty-four hours after the injection of *A. tumefaciens* cultures containing target vectors, we injected *A. tumefaciens* cultures containing BAX or Gpa2/RBP-1 into the same injection site of tobacco leaves. The results (Figure 9) showed that no necrosis was induced in the empty vector (EV) or RsVAP and RsVAP^ΔSP^ treatments. However, in the EV+BAX and EV+Gpa2/RBP-1 treatments, necrotic leaf lesions covered 75.27% and 69.81% of the injection area, respectively. In the RsVAP+BAX and RsVAP^ΔSP^+BAX treatments, the mean percentages of necrotic leaf lesions were only 1.10% and 1.00% of the injection area, respectively. In the RsVAP+Gpa2/RBP-1 and RsVAP^ΔSP^+Gpa2/RBP-1 treatments, necrotic leaf lesions covered 0.60% and 0.98% of the injection area, respectively (Figure 9C,D). After injection with BAX or Gpa2/RBP-1, the mean percentages of necrotic leaf lesions of the injection area in the RsVAP and RsVAP^ΔSP^ treatments were significantly lower than those in the EV treatment (*p* < 0.05), whereas no significant difference was detected between the former two treatments (*p* > 0.05). These results indicate that RsVAP can considerably repress BAX- and Gpa2/RBP-1-induced cell death in tobacco leaves and this repressive effect is not affected by the presence or absence of a signal peptide.

Furthermore, we extracted total protein from tobacco leaves and performed Western blot analysis with anti-BAX, anti-HA, and anti-FLAG tag mouse monoclonal antibodies as the primary antibodies. The results showed that BAX, RBP-1, RsVAP, and RsVAP^ΔSP^ proteins were all expressed correctly (Figure 9E,F).

### 2.7. Silencing of RsVAP Attenuates the Pathogenicity of R. similis on Tomatoes

**Figure 10:**
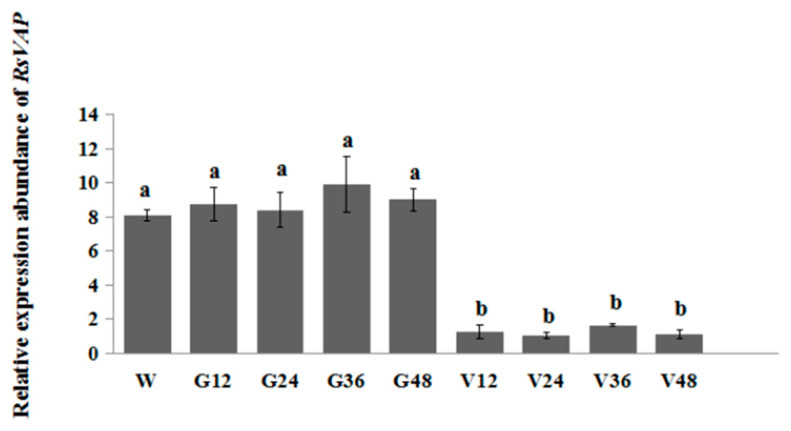
Relative expression levels of *RsVAP* in *Radopholus similis* under different RNAi treatments. W: nematodes treated with clean water; G12, G24, G36 and G48: nematodes treated with *egfp* dsRNA for 12, 24, 36 and 48 h, respectively; V12, V24, V36 and V48: nematodes treated with *RsVAP* dsRNA for 12, 24, 36 and 48 h, respectively. The *y*-axis indicates the relative expression level of *RsVAP*; bars indicate the standard errors of the mean (*n* = 3), and a, b above the bars indicate significant differences (*p* < 0.05) among groups.

We silenced the *RsVAP* gene in *R. similis* by soaking the nematodes in *RsVAP* dsRNA solution for 12–48 h. The results showed that, compared with the *egfp* dsRNA and water treatments, the relative expression levels of *RsVAP* were significantly reduced in nematodes treated with *RsVAP* dsRNA (*p* < 0.05; Figure 10). There were no significant differences in *RsVAP* expression levels between the *egfp* dsRNA and water treatments (*p* > 0.05) nor among the various periods of the *RsVAP* dsRNA treatment (*p* > 0.05). During the experimental period, the lowest expression level of *RsVAP* was observed in the *RsVAP*-dsRNA treatment at 24 h, which accounted for 13.1%, 12.1%, 12.7%, 10.7% and 11.8% of those in the water treatment and the *egfp* dsRNA treatment at 12, 24, 36 and 48 h, respectively. Based on these results, an optimal gene-silencing effect can be achieved after soaking *R. similis* in *RsVAP* dsRNA solution for 24 h.

**Figure 11:**
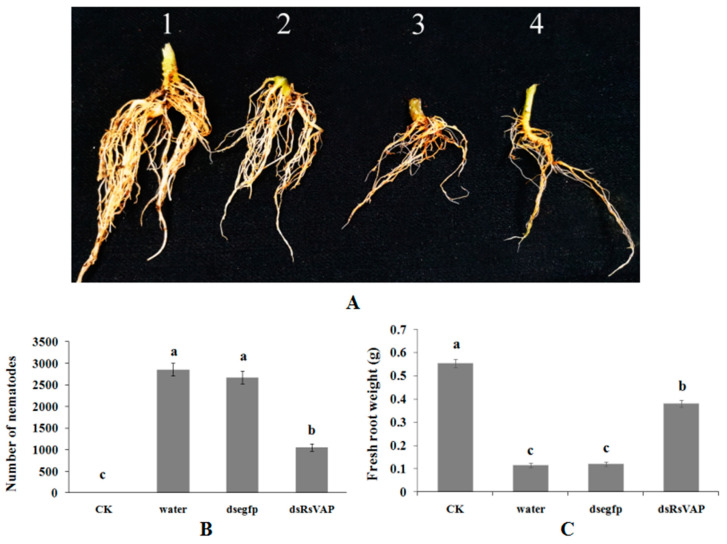
Pathogenicity and reproduction of *Radopholus similis* on tomatoes after *RsVAP* RNAi treatment. (**A**) Tomato root symptoms 30 d post-inoculation with *R. similis*, 1: healthy roots; 2: roots inoculated with *RsVAP*-RNAi nematodes; 3: roots inoculated with nematodes soaked in *egfp* dsRNA; 4: roots inoculated with nematodes soaked in water. (**B**,**C**) Number of nematodes and fresh root weight in different treatments, CK: healthy control; water: nematodes soaked by clean water for 24 h; dsegfp: nematodes soaked in *egfp* dsRNA for 24 h; dsRsvap: nematodes soaked in *RsVAP* dsRNA for 24 h. Bars indicate the standard errors of the mean (*n* = 10), and a–c above the bars indicate significant differences (*p* < 0.05) among groups.

After soaking the nematodes in *RsVAP*-dsRNA solution for 24 h, we inoculated *R. similis* into tomato roots (1000 individuals/plant); nematodes soaked in water and *egfp* dsRNA solution were inoculated as control treatments. The results showed that, compared with the non-inoculated healthy control, tomato roots inoculated with *R. similis* of different treatments showed various degrees of damage symptoms (Figure 11). In the water and *egfp* dsRNA treatments, tomato roots were severely browned, necrotic, and rotten, while the number of roots was reduced, indicating serious damage. In the *RsVAP* dsRNA treatment, the symptoms of browning and necrosis and the degree of damage were relatively mild in the tomato roots. The root fresh weight of the *RsVAP* dsRNA treatment (0.381 g) was significantly (*p* < 0.05) higher than that of the water (0.114 g) and *egfp* dsRNA (0.121 g) treatments; however, no significant difference was detected in root fresh weight between the water and *egfp* dsRNA treatments (*p* > 0.05). The number of nematodes collected from the rhizosphere was significantly (*p <* 0.05) lower in the *RsVAP*-dsRNA treatment (1051 individuals) than in the water treatment (2856 individuals) and the *egfp* dsRNA treatment (2674). No significant differences were detected in the root fresh weight or number of nematodes between the water and *egfp* dsRNA treatments (*p* > 0.05). These results indicate that the pathogenicity of *R. similis* in tomatoes is attenuated considerably after silencing *RsVAP*.

### 2.8. RsVAP Interacts with LeRabA1d in Tomato

**Figure 12:**
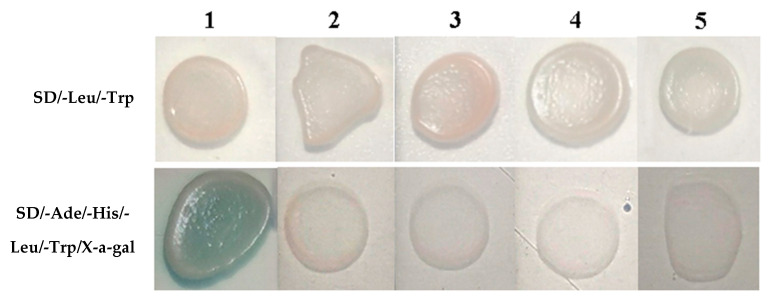
Co-transformation of yeast cells with bait vector and prey vector to verify the interaction between RsVAP of *Radopholus similis* and candidate tomato proteins. 1: pGBKT7-*RsVAP*+pGADT7-*LeRabA1d*; 2: pGBKT7+pGADT7-*LeRabA1d*; 3: pGBKT7+pGADT7; 4: pGBKT7-*RsVAP*+pGADT7-*LeOCS*; 5: pGBKT7+pGADT7-*LeOCS.*

Two genes that potentially interact with *RsVAP*, namely *LeRabA1d* and *LeOCS* (Table 1), were acquired from tomatoes using the Y2H assay. Because the two clones, respectively, contained the complete ORF sequences of *LeRabA1d* and *LeOCS*, we directly extracted the trap vectors of the two clones (pGADT7-*LeRabA1d* and pGADT7-*LeOCS*) and co-transformed them with pGBKT7-*RsVAP* into AH109 yeast competent cells to verify the interaction between the two candidate genes and *RsVAP*. The results showed that the AH109 cells co-transformed with pGADT7-*LeRabA1d* and pGBKT7-*RsVAP*, and those co-transformed with pGADT7-*LeOCS* and pGBKT7-*RsVAP*, were able to grow on SD/-Leu/-Trp plates (Figure 12). However, only the AH109 cells co-transformed with pGADT7-*LeRabA1d* and pGBKT7-*RsVAP* could grow on SD/-Ade/-His/-Leu/-Trp/X-a-gal plates and turned blue; the cells co-transformed with pGADT7-*LeOCS* and pGBKT7-*RsVAP* failed to grow on these plates. These results indicate that RsVAP interacts with LeRabA1d rather than with LeOCS in tomatoes.

**Figure 13:**
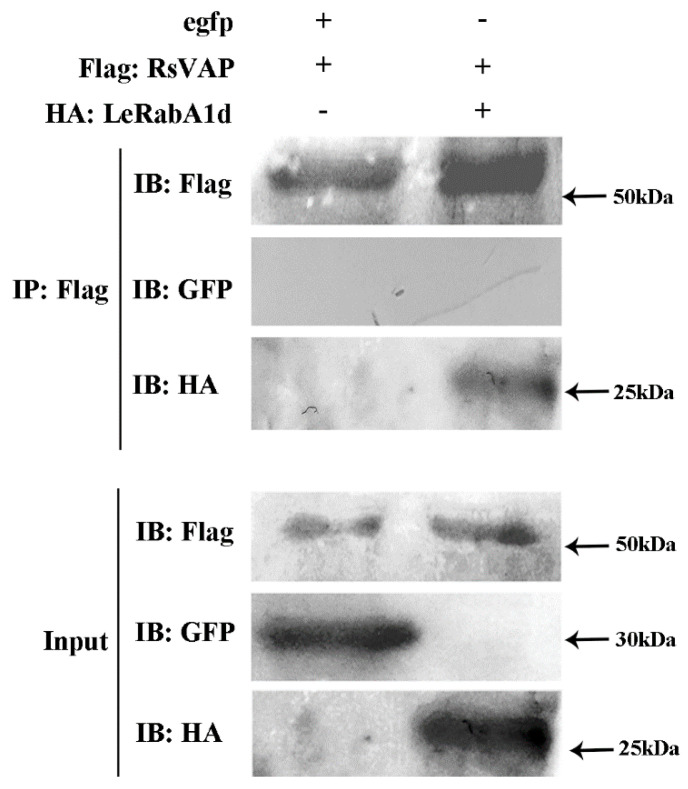
Co-immunoprecipitation assay confirming RsVAP interaction with LeRabA1d. FLAG:RsVAP and HA:LeRabA1d, as well as FLAG:RsVAP and egfp, were co-expressed in tobacco leaves. IP: Flag, tobacco leaf proteins immunoprecipitated by Anti-FLAG antibodies; input: total tobacco leaf proteins; IB: Flag, Western blot detection with anti-FLAG as the primary antibody; IB: GFP, Western blot detection with Anti-GFP as the primary antibody; IB: HA, Western blot detection with Anti-HA as the primary antibody; +, contain this vector; -, do not contain this vector.

We then performed a Co-IP assay to verify the interaction between RsVAP and LeRabA1d. The results showed that all proteins in the total protein extracted from tobacco were expressed normally. However, only LeRabA1d expression was detected in the IP samples, whereas egfp expression was not detected (Figure 13). These results confirmed that RsVAP interacts with LeRabA1d.

## 3. Discussion

In this study, we successfully cloned a novel *VAP* gene, *RsVAP*, from *R. similis* and investigated its function. We found that the full-length cDNA of *RsVAP* carries an ORF of 1089 bp, encoding a protein of 362 aa. The deduced protein contains a signal peptide of 25 aa with no transmembrane domain, and it may be secreted into the host to perform specific functions. Like other VAPs, RsVAP possesses a conserved domain of the CAP family. The results of the phylogenetic analysis showed that RsVAP is clustered with the VAPs of other plant-parasitic nematodes, including *G. rostochiensis*, *H. glycines*, *H. avenae*, and *D. destructor*, whereas the VAPs of animal-parasitic nematodes and those of free-living nematodes clustered separately. These results suggest that VAPs may play a vital role in the living habits of nematodes.

*R. similis* of various life stages and both sexes play different roles in the process of infecting the host. Both females and juveniles have infective abilities. Females also can reproduce, whereas males only can mate; males cannot infect plants because their stylet and esophageal glands are degraded [1]. Therefore, the effectors of *R. similis* should be highly expressed in juveniles and females, in contrast to their low expression in males. *Rs-eng1b* [10], *Rs-cb1* [11], *Rs-crt* [12], *Rs-far1* [13], *Rs-cps* [14], and *Rs-scp1* [15] are all important pathogenic genes of *R. similis*, which show higher expression levels in females and juveniles than in males. In the present study, we revealed that RsVAP is localized in the esophageal glands of *R. similis*, and *RsVAP* is expressed at remarkably higher levels in juveniles than in the other life stages of *R. similis*. *RsVAP* expression levels are also considerably higher in females than in eggs, with no expression in males. Furthermore, the pathogenicity and reproduction rate of *RsVAP*-silenced *R. similis* in tomato plants were substantially reduced. These results indicate that *RsVAP* plays a crucial role in host infection and pathogenesis by *R. similis*.

During long-term contact with pathogens, plants have evolved two immune response systems: PTI and effector-triggered immunity (ETI) [31]. PTI involves a series of immune responses, such as cell death, reactive oxygen species (ROS) burst, callose deposition, and upregulation of defense gene expression in plants. Defense gene expression is activated when plant cells recognize pathogen-associated molecular patterns (PAMPs) present on the pathogen’s surface when a pathogen invades the plant. The pathogen may escape from or overcome plant PTI responses through the produced effector proteins. In this context, ETI is initiated; ETI refers to the second round of immune responses activated by recognizing specific nematode effector proteins by plant cells. When compared with each other, PTI acts in a wider range but with a lower intensity; ETI acts in a narrower range but with a higher intensity and specificity. During co-evolution with plants, plant pathogens, including plant-parasitic nematodes, have evolved strategies to inhibit plant immune responses. Some studies have suggested that Ha-annexin from *H*. *avenae* represses BAX-induced cell death after its transient expression in tobacco leaves [32]; MjTTL5 from *M. javanica* interacts with AtFTRc in *Arabidopsis thaliana* to inhibit ROS burst in the host [33]; and GrCEP12 from *G. rostochiensis,* which represses cell death (induced by the ETI elicitors RBP1/Gpa2 and Rx2/CP) and ROS burst (induced by flg22) and inhibits the expression of tobacco defense genes (*NbPti5* and *NbAcre31*) [34]. Some nematodes use the post-translational modification system of plants to activate effector proteins. For example, MgGPP, an effector protein of *M. graminicola*, can be glycosylated by the post-translational modification system of plants, and glycosylated MgGPP inhibits Gpa2/RBP-1-induced cell death [35]. To date, all of the reported effectors that inhibit plant defense response are derived from sedentary plant-parasitic nematodes; whether the effectors of migratory plant-parasitic nematodes also have this function has not been reported yet. In this study, we showed that RsVAP markedly inhibits flg22-induced callose deposition after transient expression in tobacco. We also observed a significant downregulation in the expression of three PTI marker genes in tobacco, *NbPti5*, *NbGras2*, and *Nbacre31*. These findings suggest that RsVAP can inhibit flg22-induced PTI response in plants.

BAX protein is an elicitor of the hypersensitive necrotic response in plants. BAX expression in tobacco can induce a basic immune response, leading to cell death [36]. The effectors that inhibit BAX-induced cell death are thought to play a role in inhibiting the basic immune response of plants [37]. Gpa2/RBP-1 is also a set of elicitors of hypersensitive necrotic responses in plants. RBP-1 is a non-toxic protein in *Globodera pallida*, and Gpa2 is a resistance-related protein derived from plants. Co-expression of RBP-1 and Gpa2 in tobacco can trigger ETI response and induce cell death [38]. Some studies have indicated that VAPs from sedentary plant-parasitic nematodes can inhibit plant immune response. For example, the susceptibility of *A. thaliana* to *H*. *schachtii* was markedly enhanced by overexpressing *GrVAP1* from *G. rostochiensis* or *HsVAP1* and *HsVAP2* from *H*. *schachtii*. The susceptibility of *A. thaliana* overexpressing *HsVAP1* and *HsVAP2* to some other plant pathogens is also enhanced, whereas the flg22-induced immune response is inhibited [25]. Moreover, MiVAP1 of *M. incognita* and HsVAP1 simultaneously repress INF1-, Cf-4/Avr4-, and Cf9/Avr9-induced cell death; GrVAP1 and HsVAP2 also repress Cf9/Avr9-induced cell death [25], whereas HaVAP1 and HaVAP2 repress BAX-induced cell death [39]. It is worth noting that the ability of *HaVAP1*-silenced *H. avenae* to parasitize barley (*Hordeum vulgare*) was prominently enhanced, whereas the ability of *HaVAP2*-silenced *H. avenae* to parasitize wheat (*Triticum estivum*) was considerably reduced [39]. However, not all VAPs of plant-parasitic nematodes can effectively inhibit plant defense responses. To simulate esophageal gland secretions of *B. xylophilus*, Li et al. [40] used insect cells to express a large amount of BxVAP1 protein and, then, inoculated the recombinant protein into three-year-old Masson’s pine (*Pinus massoniana*). The authors observed an upregulation in the expression of the defense gene α-pinene synthase gene in the inoculated trees and evident symptoms (including plasmolysis, degradation of the nucleus and cell death) at the inoculation site, indicating that BxVAP1 can induce the host defense response and act as an immune-response elicitor. However, inoculation with BxVAP1 did not induce cavitation in pines. Therefore, the author speculated that besides *BxVAP1*, other virulence genes also function in the pathogenic process to induce cavitation in the host [40]. The different roles of *VAP* genes in various nematode species indicate that these genes may have multiple functions. In this study, we verified that, after its expression in tobacco, RsVAP repressed BAX- and RBP1/Gpa2-induced cell death. This finding indicates that similar to other VAPs from sedentary plant-parasitic nematodes, cyst nematodes, and root-knot nematodes, RsVAP also plays a role in inhibiting the defense response of plants. To our knowledge, this study is the first to report the inhibition of plant defense responses by a VAP from migratory plant-parasitic nematodes. Notably, Lozano-Torres et al. [25] showed that the VAPs of plant-parasitic nematodes could only function extracellularly, whereas Luo et al. [39] showed that either extracellular or intracellular expression of HaVAP1 and HaVAP2 could effectively repress BAX-induced cell death. Here, we constructed the vectors pCAMBIA1300:FLAG:Rs*VAP* and pCAMBIA1300:FLAG:Rs*VAP*^ΔSP^ to simulate the extracellular and intracellular expression of RsVAP. Our results indicate that irrespective of whether it is secreted to the outside of the cells, RsVAP can effectively inhibit the plant defense response; that is, RsVAP exerts its function both extra- and intracellularly.

Previous studies have shown that effector proteins secreted by plant-parasitic nematodes interact with host proteins, facilitating the infection. Therefore, we can search for host proteins that interact with the effector proteins of plant-parasitic nematodes and analyze the function of their interaction to infer the function of effector proteins. The interacting ability of some effector proteins in plant-parasitic nematodes has been identified and studied in host plants using Y2H, Co-IP, pull-down, and BIFC assays [33,41,42]. At present, relevant studies that identify host-interacting proteins have mainly focused on sedentary plant-parasitic nematodes. For example, GrVAP1 from *G. rostochiensis* interacts with the papain-like cysteine protease Rcr3pim in currant tomato, thereby inhibiting the host defense response. However, in resistant tomato plants carrying the immune receptor gene *Cf-2*, the interaction between GrVAP1 and Rcr3pim perturbs the active site of Rcr3pim, which triggers Cf-2-induced cell death [24]. Subsequent studies have shown that the VAPs of plant-parasitic nematodes can also inhibit basic immune response in plants via the following three pathways: inhibiting the activity of extracellular papain-like cysteine proteases, inhibiting the activity of extracellular subtilase-like serine proteases, and upregulating the expression of the *NPQ4* gene in chloroplasts [25]. Nonetheless, Luo et al. [39] verified that HaVAP2 from *H. avenae* interacts with the CYPRO4-like protein HvCLP rather than with papain-like cysteine proteases in barley (*Hordeum vulgare*); however, the specific mechanisms remain unclear. In this study, we identified a RsVAP-interacting protein in tomato, LeRabA1d, through Y2H and Co-IP assays. Rab proteins are members of the small GTP-binding protein superfamily and are expressed in various compartments of cells. Rab proteins, together with heterotrimeric GTP-binding proteins coupled to membrane receptors, belong to the GTP-binding proteins family; both play a vital role in cellular signal transmission.

Members of the Rab protein family are widely distributed in plants. The Rab protein family comprises eight subfamilies: RabA, RabB, RabC, RabD, RabE, RabF, RabG, and RabH [43]. This protein family has a wide range of functions and plays essential roles in plant physiological activities, such as light and hormonal regulation, growth and development, and defense response. After inoculation with *Alternaria brassicicola*, *A. thaliana* overexpressing an inherent gene, *AtRabG3b*, showed hypersensitive necrosis, indicating that AtRabG3b participates in the defense response of *A. thaliana* [44]. Overexpression of *RabE1d* in *A. thaliana* enhances plant resistance to *Pseudomonas syringae*, whereas inhibition of *RabE1d* gene expression results in abnormal plant development [45]. Moreover, *Phytophthora infestans* induces upregulation of *StRab* expression in potato, and the resistance of potato to *P. infestans* is enhanced after overexpression of *StRab* [46]. It has been proved that members of the RabA GTPases are involved in vesicular trafficking from the trans-Golgi network (TGN) to the plasma membrane and may also play a role in endocytosis [47,48], and vesicle trafficking is involved in the delivery of many immunity-related compounds [49,50,51]. Overexpression of the effector PbRxLR24 of *Phytophthora brassicae* in *A. thaliana* can significantly enhance the susceptibility of *A. thaliana* to *P. brassicae*, further studies showed that PbRxLR24 could interact with multiple RabA proteins of *A. thaliana*. This interaction will interfere with the role of the RabA proteins in the transport of vesicles from TGN to the plasma membrane, thereby inhibiting secretion process of the antibacterial proteins PR-1, PDF1.2 and possibly other defense-related compounds, and ultimately suppresses the defense response of *A. thaliana* [52].In tomato, LeRab11a is associated with fruit ripening; repressing the expression of this gene can prevent softening during tomato fruit ripening [53]. However, unlike *LeRabA1d*—the *RsVAP*-interacting gene we identified in tomato roots—*LeRab11a* is expressed in fruits, leaves, and flowers, but not in the roots. Therefore, LeRabA1d and LeRab11a are deemed to have different functions. Despite the lack of reports on the function of LeRabA1d, considering the universal functions of the Rab protein family and the specific roles of RabA proteins in vesicular trafficking, we speculate that RsVAP can affect host physiological processes, including defense response. Thus, RsVAP may benefit nematode parasitism by interacting with LeRabA1d. However, the specific function of LeRabA1d in tomatoes needs to be further explored. RsVAP, GrVAP1 and HaVAP2 have different interaction targets in their hosts, indicating that the VAPs of plant-parasitic nematodes may regulate the host defense response via different pathways.

## 4. Materials and Methods

### 4.1. Nematode and Plant Materials

The population of *R. similis* used in this study was identified and maintained by the Plant Nematode Research Laboratory of South China Agricultural University. The nematodes were cultured and propagated on carrot callus tissue at 25 °C using the method described by Fallas and Sarah [54].

Seeds of tomato *Lycopersicon esculentum* Mill. “Zhongshu-4” was purchased from Changhe Seed Co., Ltd. (Guangzhou, China). The tomato seeds were sown in pots (diameter: 15 cm) filled with autoclaved soil and, then, grown in a greenhouse with a relative humidity of 60–80%, the temperature of 26 ± 1 °C, and photoperiod of 16 h:8 h (L:D). Tomato plants (~20 cm high) were inoculated with *R. similis*. Tobacco (*Nicotiana benthamiana* L.) seeds were supplied by the Plant Nematode Research Laboratory of South China Agricultural University and grown under the same conditions as those used for tomato cultivation. Four-to-six-week-old tobacco plants were used for the experiments.

### 4.2. Total RNA and DNA Extraction from R. similis

Two groups of nematodes (~30,000 individuals per group) of mixed life stages were collected from the carrot callus tissue of cultured *R. similis*. Total RNA was extracted using Trizol reagent (Invitrogen, Carlsbad, CA, USA) following the manufacturer’s instructions, and high-quality RNA was reverse transcribed to cDNA using one-step gDNA removal and synthesis SuperMix (TransGen, Beijing, China). Genomic DNA was extracted using the phenol/chloroform extraction method [55]. The obtained cDNA and genomic DNA were stored in a refrigerator at −20 °C until use.

### 4.3. Full-Length Amplification of RsVAP

The 5′- and 3′-end sequences of *RsVAP* were amplified using the SMART RACE cDNA amplification kit (Clontech, Takara Biotechnology (Dalian) Co., Ltd., Dalian, China) following the manufacturer’s instructions. Two prime pairs, Vap-Gsp2/Vap-NGsp2 and Vap-Gsp1/Vap-NGsp1 (Supplementary Table S1), were designed for rapid amplification of cDNA ends (RACE) according to the transcript sequence [56] of a suspected *VAP* gene in the *R. similis* transcriptome. Then, two rounds of PCR amplification were performed with 5′-RACE-ready cDNA and 3′-RACE-ready cDNA as templates, respectively. The amplified fragments were sequenced, and the reads were spliced into known transcript sequences. The resultant sequences were analyzed using an open reading frame (ORF) finder (http://www.ncbi.nlm.nih.gov/gorf/orfig.cgi (accessed on 11 November 2020)) to predict the ORF of *RsVAP*. Based on the predicted results, one primer pair, FLVAP-F/FLVAP-R (Supplementary Table S1), was designed to amplify the *RsVAP* ORF. Then, PCR amplification was performed with the cDNA and DNA of *R. similis* as templates, and the amplified products were ligated into the pEASY-T cloning vector (TransGen, Beijing, China) to construct the recombinant vectors pEASY-*RsVAP* and pEASY-*gRsVAP*. Positive recombinant vectors were sequenced to obtain the full-length mRNA and genomic DNA sequences of *RsVAP*. The remaining recombinant vectors were stored in a refrigerator at −20 °C for later use.

### 4.4. Bioinformatics Analysis of RsVAP

Sequence similarity analysis of RsVAP was performed using the BLAST tool in the NCBI database (http://blast.ncbi.nlm.nih.gov/Blast.cgi (accessed on 11 October 2020)). The signal peptide of RsVAP was predicted using SignalP 4.0 (http://www.cbs.dtu.dk/services/SignalP-4.0/ (accessed on 11 October 2020)), and the transmembrane domain of RsVAP was predicted using THMMN server 2.0 (http://www.cbs.dtu.dk/services/TMHMM/ (accessed on 11 October 2020)). Based on the results of BLAST searches, 21 VAP amino acid (aa) sequences of 17 other nematode species that are closely related to RsVAP were selected and downloaded from the NCBI database (https://www.ncbi.nlm.nih.gov/ (accessed on 11 October 2020)). The selected sequences and the deduced aa sequence of RsVAP were used for multi-sequence alignment using MEGA 6.0 (http://www.megasoftware.net/ (accessed on 11 October 2020)); the phylogenetic tree was constructed using the neighbor-joining method.

### 4.5. Developmental Expression Pattern Analysis of RsVAP

Females, males, juveniles, and eggs of *R. similis* (~2000 individuals per group) were used for this analysis. Total RNA was extracted from the nematodes using the RNAprep pure micro kit (Tiangen, Beijing, China) following the manufacturer’s instructions; cDNA was synthesized using performing a reverse transcription of the extracted RNA using one-step gDNA removal and cDNA synthesis SuperMix (TransGen, Beijing, China). One specific primer pair, QVAP-F/QVAP-R (Supplementary Table S1), was designed for quantitative reverse-transcription PCR (RT-qPCR) according to the full-length mRNA sequence of *RsVAP*, and *eif5a* [57] was used as an internal reference gene. RT-qPCR was performed using AceQ qPCR SYBR Green Master Mix (Vazyme, Nanjing, China) on the Bio-Rad CFX-96 system (Bio-Rad Laboratories, Hercules, CA, USA). Relative gene expression levels were calculated using the 2^−ΔΔct^ method [58]. The experimental data were subjected to one-way analysis of variance (ANOVA) using GraphPad Prism (https://www.graphpad.com/scientific-software/prism/ (accessed on 20 October 2020)), and a multiple comparison test was conducted at 0.05 significance level. The experiment was performed using three biological replicates.

### 4.6. In Situ Hybridization

A 294-bp fragment was selected for probe synthesis according to the full-length ORF sequence of RsVAP. Specific primers for the sense probe (ISVAP-T7F/ISVAP-R) and the antisense probe (ISVAP-F/ISVAP-T7R) were designed based on the sequence of the selected fragment (Supplementary Table S1). The pEASY-*RsVAP* vector was used as a template for PCR amplification. The purified PCR products were labeled with the DIG RNA labeling mix (Roche, Basel, Switzerland) to synthesize the sense and antisense probes. In situ hybridization was performed using the method described by de Boer et al. [59]. The results were observed and photographed using a Nikon Eclipse 90i microscope (Nikon Corp., Chiyoda, Tokyo, Japan).

### 4.7. Southern Blot Analysis

A 251-bp fragment was selected for probe synthesis based on the ORF sequence information of RsVAP. A digoxigenin-labeled Southern blot probe was synthesized by PCR using the specific primers VAP-S-F/VAP-S-R (Supplementary Table S1) and the PCR DIG probe synthesis kit (Roche, Basel, Switzerland) with pEASY-*RsVAP* as a template. Two genomic DNA samples of *R. similis* (10 μg each) were single-digested with the restriction enzymes SacI and NcoI (Thermo, Waltham, MA, USA). The pEASY-*RsVAP* plasmid, single-digested with the same restriction enzymes, was used as a control. Southern blot analysis was conducted using the DIG high prime DNA labeling and detection starter kit I (Roche, Basel, Switzerland), following the manufacturer’s instructions.

### 4.8. Inhibition Analysis of RsVAP on flg22-Induced PTI

Two primer pairs with FLAG tag, PL468F/PL468R and PL469F/PL468R, were used to amplify the FLAG:*RsVAP* fragment with a FLAG tag at the 5′ end and the FLAG:*RsVAP*^ΔSP^ fragment containing no signal peptide, respectively. The pEASY-*RsVAP* plasmid was used as a template. The PCR products were ligated into the pCAMBIA1300 vector to construct the recombinant vectors pCAMBIA1300:FLAG:Rs*VAP* and pCAMBIA1300:FLAG:Rs*VAP*^ΔSP^, which were then transformed into *Agrobacterium tumefaciens* EHA105 for later use.

#### 4.8.1. Callose Deposition Analysis

*A. tumefaciens* cultures containing pCAMBIA1300:FLAG:Rs*VAP*, pCAMBIA1300:FLAG:Rs*VAP*^ΔSP^, and pCAMBIA1300:*egfp* were grown to an optical density at 600 nm (OD_600_) of 1.0–1.5. Then, the cultures containing target vectors were injected on the adaxial surface of tobacco leaves in 4-to-6 week-old seedlings. After injection, the seedlings were cultivated in the dark at 28 °C for 48 h. Subsequently, leaf-tissue samples of appropriate size (~1 cm^2^) were taken and soaked in 10 μM flg22 solution for 3 h. Callose staining was performed using the method described by Fabro et al. [60]. The results were observed using a Nikon Eclipse Ni microscope (Nikon Corp., Chiyoda, Tokyo, Japan). The experiment was performed using three biological replicates.

#### 4.8.2. Defense Gene Expression Analysis

*A. tumefaciens* cultures containing target vectors were injected into tobacco leaves as described previously for the callose deposition analysis. The injected tobacco seedlings were cultivated in the dark at 28 °C for 48 h. Subsequently, leaf tissue samples were taken and soaked in 10 μM flg22 solution for 3 h. Total RNA was extracted from tobacco-leaf samples using the HiPure plant RNA mini kit (Magen, Shanghai, China). Total RNA was then reverse transcribed into cDNA using one-step gDNA removal and cDNA synthesis SuperMix (TransGen, Beijing, China). RT–qPCR assay was performed on the Bio-Rad CFX-96 system (Bio-Rad Laboratories, Hercules, CA, USA) to quantify the relative expression of three PTI marker genes of tobacco—*NbPti5* [30], *NbGras2* [30], and *Nbacre31* [30]; *NbEF1* [61] of tobacco was the internal reference gene. Tobacco leaves soaked in flg22 after egfp injection (as previously described) and leaves soaked in clean water were used as controls. The experiment was performed using three biological replicates.

### 4.9. Inhibition Analysis of RsVAP on Immune Elicitor-Induced Cell Death

Two sets of immune elicitors, BAX and Gpa2/RBP-1, were used to test the inhibition of cell death by RsVAP. *Agrobacterium tumefaciens* cultures containing pCAMBIA1300:FLAG:Rs*VAP*, pCAMBIA1300:FLAG:Rs*VAP*^ΔSP^, and pCAMBIA1300:*egfp* (OD_600_ = 1.0–1.5) were injected into tobacco leaves; the seedlings were cultivated at 28 °C for 24 h. Afterward, *A. tumefaciens* cultures containing the immune elicitors (OD_600_ = 1.0–1.5) were injected into the adaxial surface of tobacco leaves. The number of injection points was 20 per treatment. After injection, the seedlings were cultivated at 28 °C for 3–6 days. The necrosis of tobacco leaves was examined and photographed using a Canon camera DS126181 (Canon, Tokyo, Japan). The necrotic index was estimated using the method described by Gilroy et al. [62].

### 4.10. Western Blot Analysis

After injection with immune elicitors, tobacco leaf samples were collected from the injection site, and total protein was extracted using the plant protein extraction kit. Western blot analysis was performed following the method described by Chen et al. [35]. Specifically, anti-BAX, anti-HA and anti-FLAG mouse monoclonal antibodies (TransGen, Beijing, China) were used to detect BAX protein, RBP-1 protein, and RsVAP and RsVAP^ΔSP^, respectively. Goat anti-mouse IgG and HRP conjugate (TransGen, Beijing, China) were used as secondary antibodies for all Western blot analyses. Metal enhanced DAB substrate kit (Solarbio, Beijing, China) was used for protein staining.

### 4.11. RNAi and Pathogenicity Assay

The sense- and antisense-strand templates of *RsVAP* dsRNA were obtained by PCR amplification using two specific primer pairs, VapiT7-F/Vapi-R and Vapi-F/VapiT7-R (Supplementary Table S1), with the pEASY-*RsVAP* plasmid as a template. The dsRNA of *RsVAP* was synthesized using the method described by Huang et al. [15]; the dsRNA solution obtained was frozen at −80 °C until use. The same method was used to synthesize *egfp* dsRNA with two specific primer pairs, egfpiT7-F/egfpi-R and egfpi-F/egfpiT7-R (Supplementary Table S1).

Approximately 500 *R. similis* individuals of mixed life stages were collected and soaked in 50 μL of *RsVAP* dsRNA solution (2 μg/μL). The duration of treatment was set to 12, 24, 36 and 48 h. Nematodes soaked in *egfp* dsRNA solution (2 μg/μL) for the same periods were used as negative controls, and nematodes soaked in clean water were used as blank controls. After treatment, all nematodes were washed three times with clean water, followed by RNA extraction and reverse transcription. RT–qPCR was performed to quantify the relative expression levels of *RsVAP* in the different treatments. The experiment was performed using three biological replicates. The optimal soaking period for gene silencing was selected for a subsequent pathogenicity test.

After soaking in *RsVAP* dsRNA solution, *egfp* dsRNA solution, or clean water, *R. similis* individuals of mixed life stages were inoculated into the roots of tomato plants (~20 cm high). Each plant was inoculated with approximately 1000 nematodes. The inoculated plants were cultivated in an incubator at constant temperature (27 °C), relative humidity (75%), and photoperiod (12 h:12 h, D:L). Thirty days later, the damage to the tomato plants was observed. The number of nematodes in the soil and plant roots was counted, and the fresh-root weights were measured. The experiment was performed twice, and five biological replicates were used per experiment.

### 4.12. Screening and Verification of RsVAP-Interacting Proteins in Tomato

The *RsVAP*^ΔSP^ fragment containing no signal peptide was amplified using the specific primer pair PL524FXIN/PL524RXIN (Supplementary Table S1), with the pEASY-*RsVAP* plasmid as a template. The PCR products and empty vector pGBKT7 were double-digested with BamHI and PstI and then ligated to construct the bait vector pGBKT7-*RsVAP*. Next, pGBKT7-*RsVAP* was transformed into AH109 yeast competent cells (Weidibio, Shanghai, China) and tested for toxicity and self-activation using the Matchmaker™ gold yeast two-hybrid system (Clontech, Dalian, China) according to the manufacturer’s instructions.

The Y2H assay was performed using the Matchmaker™ Gold Yeast Two-Hybrid System (Clontech, Dalian, China) with AH109 yeast cells carrying the bait vector and tomato root library strains maintained in our laboratory. Monoclonal colonies of yeast were selected from SD/-Ade/-His/-Leu/-Trp plates, streaked onto SD/-Ade/-His/-Leu/-Trp/X-α-Gal plates, and incubated in the dark at 30 °C for 3–5 days. If colonies grew on the plates and turned blue, there was a possible interaction between the target and the prey proteins. Blue colonies were then selected and amplified by PCR using the universal primer pair pGADT7-F/pGADT7-R (Supplementary Table S1). The amplified products were sequenced, and the sequencing results were aligned using BLASTx in the NCBI database.

Yeast plasmids were extracted from blue colonies using the HiPure yeast plasmid mini kit (Magen, Shanghai, China) and transformed into *Escherichia coli* DH5α competent cells. The pGADT7-*candidate gene* vector was selected according to an ampicillin resistance marker carried by the vector pGADT7 in the library strains. The bait vector pGBKT7-*RsVAP* and the vector pGADT7-*candidate gene* were co-transformed into AH109 yeast competent cells (Weidibio, Shanghai, China); the transformed-cell suspension was spread onto SD/-Leu/-Trp and SD/-Ade/-His/-Leu/-Trp/X-α-Gal plates. The growth of yeast colonies on the plates was observed after incubation in the dark at 30 °C for 3–5 days.

To verify the interaction between RsVAP and its potential interacting proteins, a co-immunoprecipitation (Co-IP) assay was performed using a tobacco transient expression system. RNA was extracted from tomato leaves using a HiPure plant RNA mini kit (Magen, Shanghai, China). PCR amplification was performed using the specific primer pair PM247F/PM247R (Supplementary Table S1) to obtain the *LeRabA1d*:HA fragment. The *LeRabA1d*:HA fragment was ligated into the pCAMBIA1300 vector to construct the pCAMBIA1300:*LeRabA1d*:HA vector, then transformed into *A. tumefaciens* EHA105. *A. tumefaciens* cultures containing the pCAMBIA1300:*LeRabA1d*:HA and pCAMBIA1300:FLAG:Rs*VAP*^ΔSP^ vectors were grown to OD_600_ = 1.0–1.5 and mixed in equal volumes. The culture mixture was injected into the tobacco leaves for co-expression. Three days later, the total protein of the tobacco leaves was extracted using the plant protein extraction kit (CWBiotech, Beijing, China), and a Co-IP assay was performed using the Capturem^TM^ IP and Co-IP Kit protocol-At-A-Glance (Takara, Dalian, China) following the manufacturer’s instructions. Protein expression in the IP and Input samples was detected using anti-HA, anti-flag, and anti-GFP mouse monoclonal antibodies (TransGen, Beijing, China).

## Figures and Tables

**Table 1:** Functional annotation of candidate tomato proteins interacting with RsVAP.

Clone	Gene Name	Amino Acid Sequence Coverage	Amino Acid Sequence Identity
1	Ras-related protein *RabA1d*	100%	99.54%
2	Oligosaccharyltransferase complex subunit *CG9662*	100%	100%

## Data Availability

The data presented in this study are available on request from the corresponding author.

## Supplementary Materials

The following are available online at https://www.mdpi.com/article/10.3390/ijms22094782/s1, Figure S1: Prediction of signal peptide and transmembrane domainin RsVAP of *Radopholus similis*., Table S1: Primers used in this study.
