# Supplementary material for: A Venom Allergen-Like Protein, RsVAP, the First Discovered Effector Protein of Radopholus similis That Inhibits Plant Defense and Facilitates Parasitism
Source: Int J Mol Sci. 2021 Apr 30;22(9):4782. doi: 10.3390/ijms22094782 (PMC8125365; doi:10.3390/ijms22094782)
Supplement: Supplementary file 1 [file ijms-22-04782-s001.zip › supplementary material/data and sequences/Sequences of RsVAP.pdf]

Full-length cDNA sequence of *RsVAP* （The red part is the ORF of *RsVAP*)

ACATGGGGCATTTCTATCTTCTTCTAAATTCTCAACCTGCTGGCCAAAACCCCCCAAAA
GCCCAAATCTGAAAATCTGA**ATG**AGTCCGTTTCGCTTCTTCTGCCTGGGAGCAGTCATT
TTGACTGGAGCGACCATGTTTCTGCTGTCAGAAGCGAGTGGGGGAGGAATGGAAAAG
GAGATCAGAGAGCTCGTCGAGAAGCTGAATGCGGAGCACCTGCTCAAGCATCACAAG
GGCCCATCCGGCGCCAGAATTCGAATTGAACGCGGCCGATCCGAGCAACGGGGCAGC
CACAGCAGAAGTGGTTCACGCTCAAGATCGGGGTCAAGGGGCAGCGAAAACAGGTCA
TCATCGAGAAGCGGCAGCCGCAGGTCAAGTTCGAGAGGGAGCAGATCTAGTACTGGT
TCGAGATCGGCCTCTAATAAGTCAAGGTCATCGAGGAGCGGCGGCAACAGATCATCAC
ACAGTTCACATCGCCATTCGAGCCATGGACATACGACGGCATCTTCTTCAAAAAATAAA
TCACACCATCGATCCAAAAAAGGTGCGCTGACCAGTGCGGAGCGTAAAGCGGTGCTG
GATGCGCACAACAATTACCGCTCGACGTTGGCTCGTGGAAAGGCCCGCAACAAGGAC
GGCAAGATGCTCCCGACAGCAGCGAACATGGTGAAGTTGCGCTACAATGTGGAGCTG
GAAAAGTACGCGGACGCGTGGGCCAAGCGCTGCTGGTTTGAGCATACGGAAAAATAT
GCGCACTCGACAAAACAGAAGGCCAGATACGAGGACAGTGGTGGCTACGGCGAGAAT
TTGGCCGTTGTGTATCAGAAGGTAAAAGCCGCAGCCCTCAAACAGGCCAGCCTCATAT
TTTGGGATGAGTTGAAGGAGTGCGGGATTGACCGCAACACTTTGGTGCACACCCGCA
AGTGTATGTTGGGCCATTGGACGCAAATGGCGTGGGCAACCAGCACAGAGCTGGGATG
CGCGGTGGCCCACTGTGACTTGATTCGCGAAAAGAACGGAGCAGAATATAAAGACGG
CGTATACATTGTGTGCCAATACACACCAAGTGGCAACTGGCAAGGCCAGCCCGTCTAC
AAAAGTGGGCCCGTGTGCTCGGAATGTCCGAGCGGCTCCCACTGCGAGCGCAGGACA
GGATTGTGCGTCTAC**TGA**AAAAGAAAATATAGCCAAATGGATGGGGAGGAAAATTCAC
TGGGCTCTTTTCGGTGTTCCAAACTGATACAAATTTTGCAACTCATAAAATGATGAAAC
GAAAAAAAAAAAAAAAAAAAAAAAAAAAAAAAGT

Full-length DNA sequence of *RsVAP*（The red part are the introns of *RsVAP*)

ATGAGTCCGTTTCGCTTCTTCTGCCTGGGAGCAGTCATTTTGGCTGGAGCGACCATGTT
TCTGCTGTCAGAAGCGAGTGGGGGAGGAATGGAAAAGGAGATCAGAGAGCTCGTCGA
GAAGCTGAATGCGGAGCACCTGCTCAAGCATCACAAGGGCCCATCCGGCGCCAGAAT
TCGAATTGAACGCGGCCGATCCGAGCAACGGGGCAGCCACAGCAGAAGTGGTTCACG
CTCAAGATCGGGGTCAAGGGGCAGCGAAAACAGGTCATCATCGAGAAGCGGCAGCCG
CAGGTCAAGGTCATTAAAAAGCAGAAAAGAGAGGACGTCAATTCTTTTTAGCACCCCA
TAGTTTTAGCCTAAGACGAACTTATTCTTAGCTTAACTTAGGCTTAATCCAAGTCTACGC
CTAACGTCTGATGCGTTTTAGCCTAAGCTTAAGAAAAGTGAAAATAATTGTTTTGGTGG
GTAAAAAATATTATGCCGAACACAGAGCCTTGCCTACACAACGCCAAACATGGCACAT
TTTGTCCCAAGTCAATCATCATTCTCAAATCCCAGTTCGAGAGGGAGCAGATCTAGTAC
TGGTTCGAGATCGGCCTCTAATAAGTCAAGGTCATCGAGGAGCGGCGGCAACAGATCA
TCTCACAGTTCACATCGCCATTCGAGCCATGGACATACGACGGCATCTTCTTCAAAAAA
TAAATCACACCATCGATCCAAAAAAGGTAGTCGTGTTCGCTCAACACATATAAGGACG
CGACATTTGAGGATTTAGGCCGATCCCGGATTGGTTAATTGAATCATTTTGGGTGTAAAA
TTGATCATTATTTCTCCTCAAAATATCCTCAAATATCCGTCCGCAAATGTCGGCTAACTGT
AGACAATAATTTGAAGAGCAATTTCCAAACGGTGCTCTTAGATGCGGTACAAGTCGTTT
GGGCGACGGGGGCTTTAGACCAGTTCGGGCCACGTTATGGCCAAAAAAGTCCAAAAA
TCCCTGATTTTGTCCAAACATTTGGCCATATCTTGGCCAATTTTCGGGATATCCAATAATT

TATTAGACCAATGGATAGAGCACACTTTGGGCTTTCCAATGGCATTTTATTTAAATTTTT
AATCTAAATCTTGACCGAGTTATGGTTAATCAAAAGCCTCCGTCGCCCAAACGATATGC
ATCGCTCTAGGTGCGCTGACCAGTGCGGAGCGTAAAGCGGTGCTGGATGCGCACAAC
AATTACCGCTCGACGTTGGCTCGTGGAAAGGCCCGCAACAAGGACGGCAAGATGCCC
CCGACAGCAGAGAACATGGTGAAGTTGCGCTACAATGTGGAGCTGGAAAAGTACGCG
GACGCGTGGGCCAAGCGCTGCTGGTTTGAGCATACGGAAAAATATGCGCACTCGACAA
AACAGAAGGCCAGATACGAGGACAGTGGTGGCTACGGCGAGAATTTGGCCGTTGTGTA
TCAGAAGGTAAAAGGTGCGCAGAAAATGGAACCAAATTTATAAAATAAATTTGTAATTA
AAATCAGAGCAGGAATGTGTGCCGGGATTAAGCATTGAGAGAGGATCAGAACTCAGA
ACCTTTCGATGAAAGCCATTTGAAACGAAATGCTAAAACAAGAAAATATCAACGTCTA
AACACAACCAATCCGAACAGACAAGTTTCAGTGCCTGGGGGGTTTGAGAGGTCATAA
CTCATAACTCAGCCAAGTTTAAGGCTAAGCAAGTGAATGAGATGTCATTGGAAAGCTC
AAAGTGTACACTATCAATGGCTTTATTCAGCTTTCCGGCTCTCTCAGCATTGGCCAAGTT
ATCGCCAAATGTTTGGAAAAATCGGCTAATTTCCGTTTTTAGGCCATAACTTGTTCAAA
AGCCGGACCAATCAATTCAGTTTTACCCATTTTTGTAGAGCTTCCACAGATCTGTCCAA
TGAGTCCAAGAACTCTTTCTTAGCTTCATTCTTTGCCGAGTTGTCTCCCTTCAACCGTC
CTAGCACTGAAATGTCCCTTCTTTTAAAGTATACATATAATTCCGGCATATTCCTGTTCCC
TCCGATTTAATGTGCTTGGAGTGTTTTCTTTTTGTTCTAGCCGCAGCCCTCAAACAGGC
CAGCCTCATATTTTGGGATGAGTTGAAGGAGTGCGGGATTGACCGCAACACTTTGGTG
CACACCCGCAAGTGTATGTTGGGCCATTGGACGCAAATGGCGTGGGCAACCAGCACA
GAGCTGGGATGCGCGGTGGCCCACTGTGACTTGATTCGCGAAAAGAACGGAGCAGAG
TATAAAGACGGCGTATACATTGTGTGCCAATACACACCAAGGTTAGTTCCTCAAACAAG
ATGTTGGGGGCTCTACTGCGGATTTAGAGCATCCAATCAAACTTTTTGTCCGTTTGTTC
AATCAATCTTAAGCTTGGCTTACACTTTGGGGCTTTTGCAAGGCTACTACTTTGGTATAT
TTTTTAGCAGAACGACCCTAGAGTAAGTTGGTTGTTCAGTCTTTTTTGTCCGGTTCGTT
CACTCACTCTTCTGAGGCTTCAGCCCGGTTGGGCGATAATTAAATGAACAACGCACAA
TGGGAAAGGCAGAGGCTTTAAAGGGAGCAAGAGCTTAGTCGGTGGGTGAAACCTTCC
AAACCTAGCAAGACTAAGCCTAGTAAGACCCAAAATGTAGTCAAGCTTTTAAAATCGA
TTGGAAATCCAAAAGTTGATTGGACAGCTAAACCGTGAGAGAGTGGATTAATTCCCTG
CCAAAAGTGGTTGAAAAAGCTACCAAATAAATGTTTCTTCAACCCAAATAGTGCCCCC
ACCAAGGACAGCAGTAAATAGCTGATTCGAAGCGTCTCTCTAAGAGCTGAATGGAATG
GTTCCAAAATTAGGCCGATCTGACCATTCGGAAAAAAGTTATTCAGCGTCAAAAATTCA
TGCAGACATTTGGGAGCTTTTCAAAAACCATTTTTGAAAGTTAGCGAATCCTAAACCGT
AATAGAGCCCCAAGACTCTAGTCTGGACGTCTGGAGCTTTAGCCAATGGGAGGGACTT
TTATTCACAACAATTCTAAAGCGCTTTCTTTTTGTCAAAGTGGCAACTGGCAAGGCCAG
CCCGTCTACAAAAGTGGGCCCGTGTGCTCGGAATGTCCGAGCGGCTCCCACTGCGAGC
GCAGGACAGGATTGTGCGTCTACTGA
