# Supplementary material for: A Venom Allergen-Like Protein, RsVAP, the First Discovered Effector Protein of Radopholus similis That Inhibits Plant Defense and Facilitates Parasitism
Source: Int J Mol Sci. 2021 Apr 30;22(9):4782. doi: 10.3390/ijms22094782 (PMC8125365; doi:10.3390/ijms22094782)
Supplement: Supplementary file 1 [file ijms-22-04782-s001.zip › supplementary material/Supplementary Tables and figures .pdf]

Table S1 Primers used in this study

Primer name	Primer sequence (5'–3')
Vap-Gsp2	AAGTGGACCCGTGTGCTCGGAATG
Vap-NGsp2	GGGCTCTTTTCGGTGTTCCAAACTGA
Vap-Gsp1	CCGTAGCCACCGATGTCCTCGTATTT
Vap-NGsp1	CGTACTTTTCCAGCTCCACATTGTAGCG
FLVAP-F	ATGAGTCCGTTTCGCTTCTTCTGCC
FLVAP-R	TCAGTAGACGCACAATCCTGTCCTG
QVAP-F	GGGAGGAATGGAAAAGGAGA
QVAP-R	ATCTTGAGCGTGAACCACTT
Qeif5a-F	AGAGACGAGGATGAGTTT
Qeif5a-R	GAGAGAGGAGAATTTGTTGAT
ISVAP-T7F	TAATACGACTCACTATAGGGCGTTGGCTCGTGGAAAGG
ISVAP-R	GGTCAATCCCGCACTCCT
ISVAP-F	CGTTGGCTCGTGGAAAGG
ISVAP-T7R	TAATACGACTCACTATAGGGGGTCAATCCCGCACTCCT
VAP-S-F	GAGCGTAAAGCGGTGCTGGAT
VAP-S-R	GCCAAATTCTCGCCGTAGCC
PL468F	CGCGTCGACATGGATTACAAGGATGACGACGATAAGAGTCCGTTTCG CTTC
PL468R	GTTCTGCAGTCAGTAGACGCACAA
PL469F	CGCGTCGACATGGATTACAAGGATGACGACGATAAGAGTGGGGGAG GAATG
QNbPti5-F	CCTCCAAGTTTGAGCTCGGATAGT
QNbPti5-R	CCAAGAAATTCTCCATGCACTCTGTC
QNbAcre31-F	AATTCGGCCATCGTGATCTTGGTC
QNbAcre31-R	GAGAAACTGGGATTGCCTGAAGGA
QNbGras2-F	TACCTAGCACCAAGCAGATGCAGA
QNbGras2-R	TCATGAGGCGTTACTCGGAGCATT
QNbEF1-F	AGAGGCCCTCAGACAAAC
QNbEF1-R	TAGGTCCAAAGGTCACAA
VapiT7-F	TAATACGACTCACTATAGGGCGTTGGCTCGTGGAAAGG
Vapi-R	GGTCAATCCCGCACTCCT
Vapi-F	CGTTGGCTCGTGGAAAGG
VapiT7-R	TAATACGACTCACTATAGGGGGTCAATCCCGCACTCCT
egfpiT7-F	TAATACGACTCACTATAGGGTTCAAGTCCGCCATGCCCGAA
egfpi-R	CATGTGATCGCGCTTCTCGTT
egfpi-F	TTCAAGTCCGCCATGCCCGAA
egfpiT7-R	TAATACGACTCACTATAGGGCATGTGATCGCGCTTCTCGTT
PL524FXIN	CGCGGATCCGTATGAGTGGGGGAGGAATGGAA
PL524RXIN	GTTCTGCAGTCAGTAGACGCACAATCC
pGADT7-F	GGAGTACCCATACGACGTACC
pGADT7-R	TATCTACGATTCATCTGCAGC
PM247F	CGCGTCGACATGGCTGGGTACAGA
PM247R	TGGCTGCAGTTAAGCGTAATCTGGAACATCGTATGGGTACATGGATG AACAGCAACC

The T7 sequence is underlined.

MSPFRFFCLGAVILTGATMFLLSEASGGGMEKEIRELVEKLNAEHLLKHHKGP
SGARIRIERGRSEQRGSHSRSGSRSRSGSRGSENRSSSRSGSRRSSSRGSRSSTG
SRSASNKSRSSRSGGNRSSHSSHRHSSHGHTTASSSKNKSHHRSKKGALTSAE
RKAVLDAHNNYRSTLARGKARNKDGKMLPTAANMVKLRYNVELEKYADAW
AKRCWFEHTEKYAHSTKQKARYEDSGGYGENLAVVYQKVKAAALKQASLIF
WDELKECGIDRNTLVHTRKCMLGHWTQMAWATSTELGCAVAHCDLIREKNG
AEYKDGVYIVCQYTPSGNWQGQPVYKSGPVCSECPSGSHCERRTGLCVY

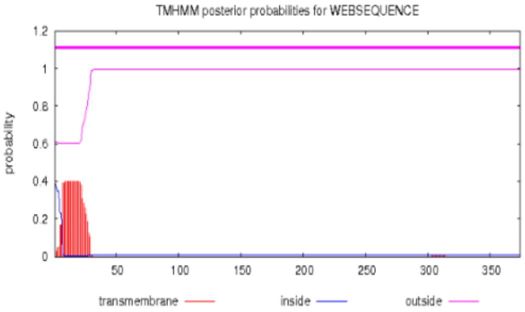

A B

Figure S1 Prediction of signal peptide and transmembrane domainin RsVAP of *Radopholus similis.* A: The deduced amino acid sequence of RsVAP comprising a signal peptide; B: Transmembrane domain prediction of RsVAP.
