# Supplementary material for: A Venom Allergen-Like Protein, RsVAP, the First Discovered Effector Protein of Radopholus similis That Inhibits Plant Defense and Facilitates Parasitism
Source: Int J Mol Sci. 2021 Apr 30;22(9):4782. doi: 10.3390/ijms22094782 (PMC8125365; doi:10.3390/ijms22094782)
Supplement: Supplementary file 1 [file ijms-22-04782-s001.zip › supplementary material/western-blot/annotation.pdf]

Western blots of Figure 9

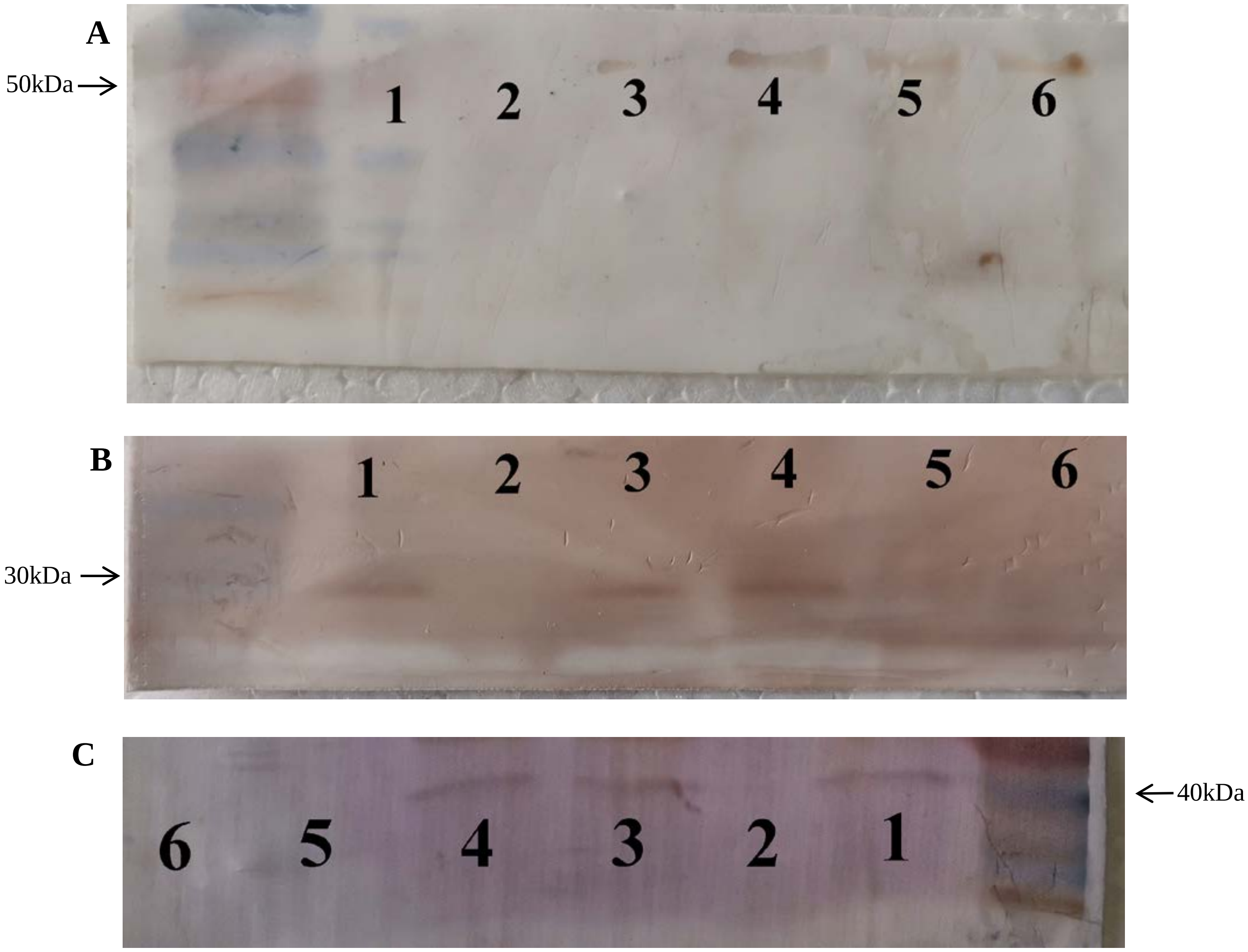

A: Detection of RsVAP/RsVAP$^{\Delta SP}$ (Flag) expression by Western blot analysis; B: Detection of BAX expression by Western blot analysis; C: Detection of RBP-1 (HA) expression by Western blot analysis. 1: pCAMBIA1300→24hours→BAX; 2: pCAMBIA1300; 3: RsVAP→24hours→BAX; 4: RsVAP$^{\Delta SP}$→24hours→BAX; 5: RsVAP; 6: RsVAP$^{\Delta SP}$. B, F: 1: pCAMBIA1300→24hours→Gpa2/RBP-1; 2: pCAMBIA1300; 3: RsVAP→24hours→ Gpa2/RBP-1; 4: RsVAP$^{\Delta SP}$→24hours→ Gpa2/RBP-1; 5: RsVAP; 6: RsVAP$^{\Delta SP}$

Western blots of Figure 13

IP: Flag-IB: Flag

50kDa

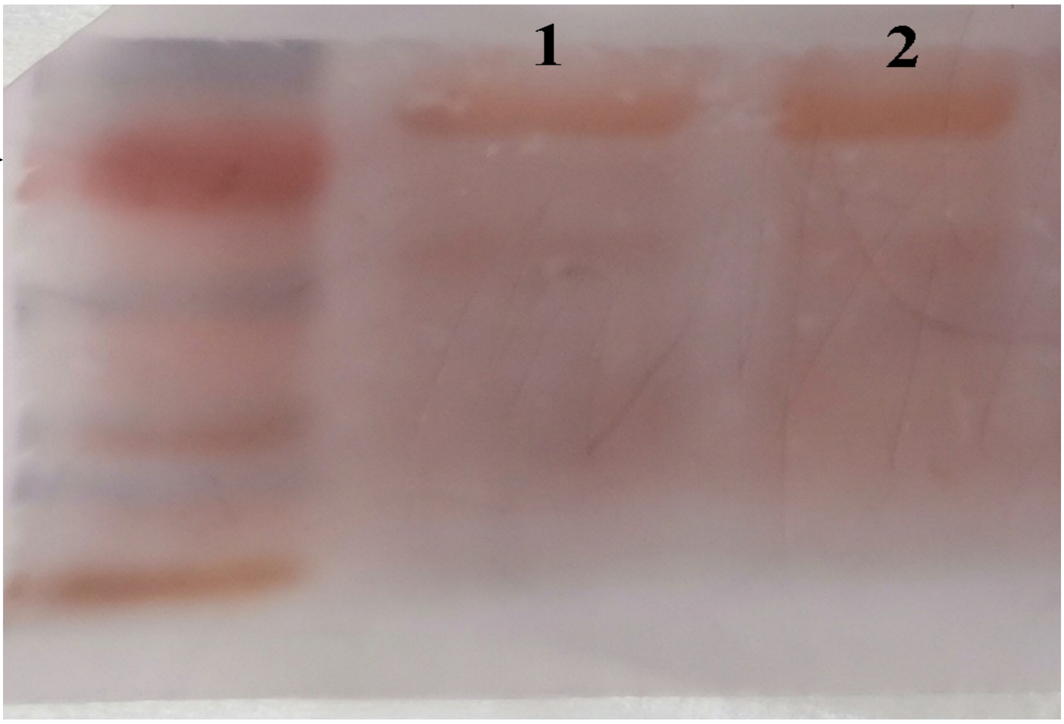

IP: Flag-IB: GFP

30kDa

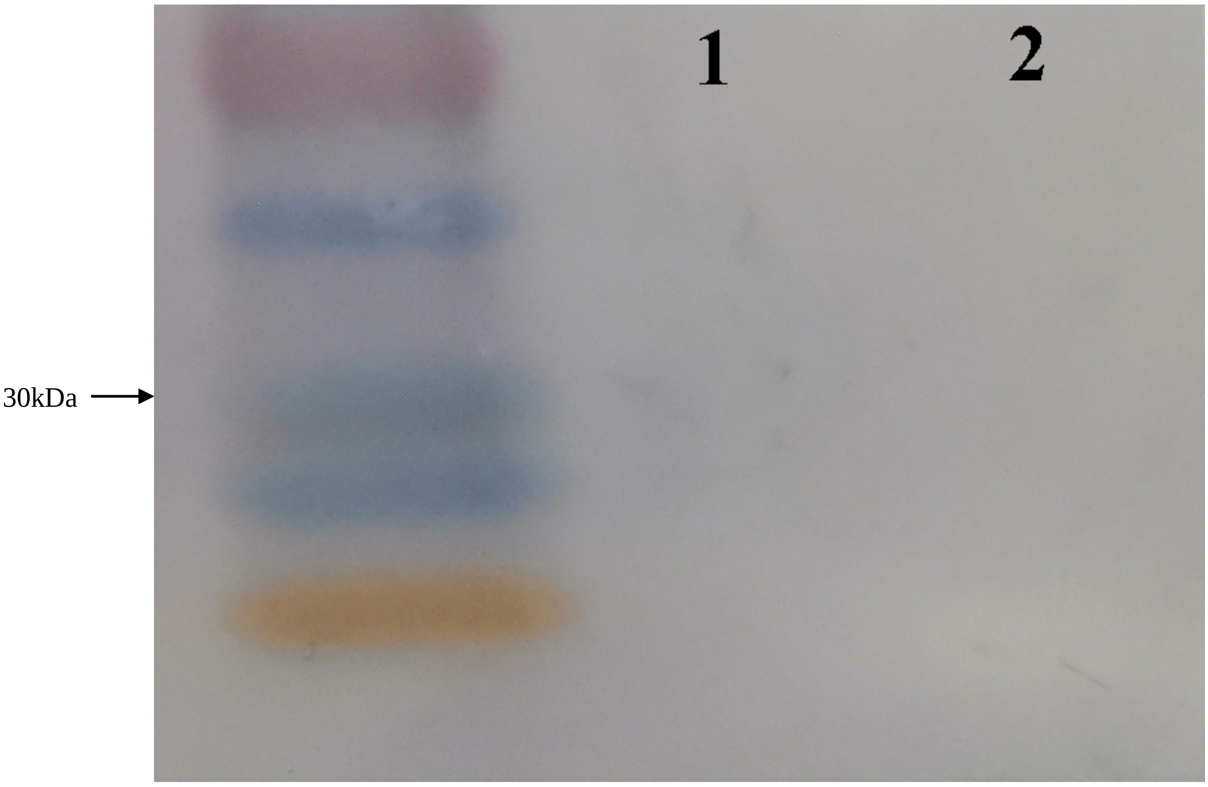

IP: Flag-IB: HA

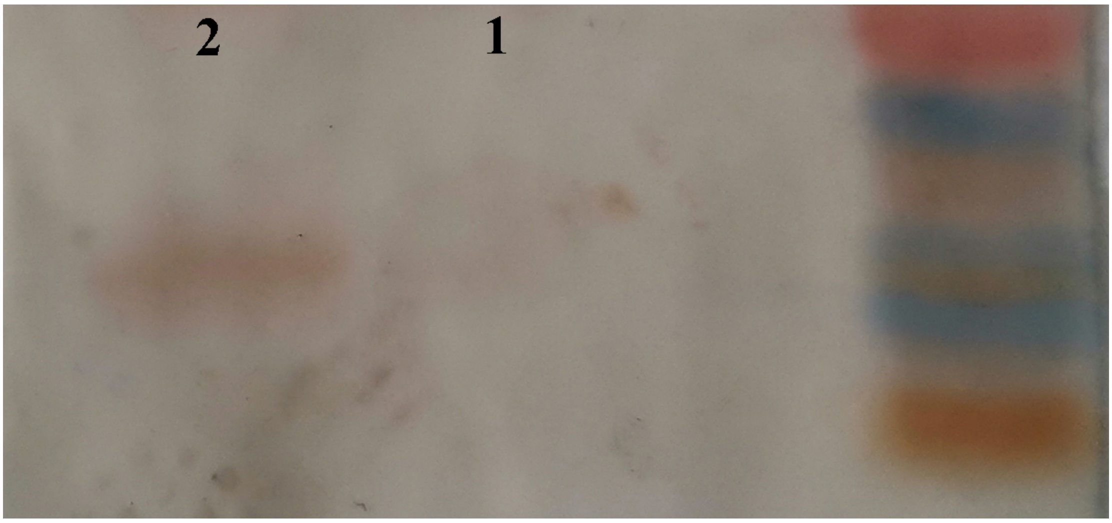

25kDa

Input-IB: Flag

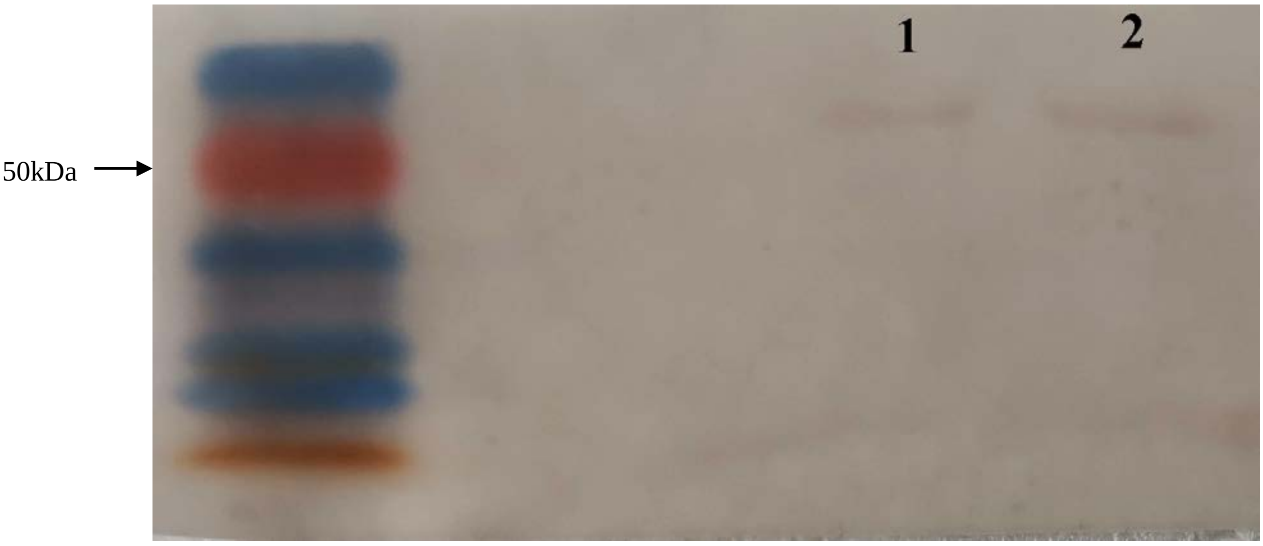

50kDa

Input-IB: GFP

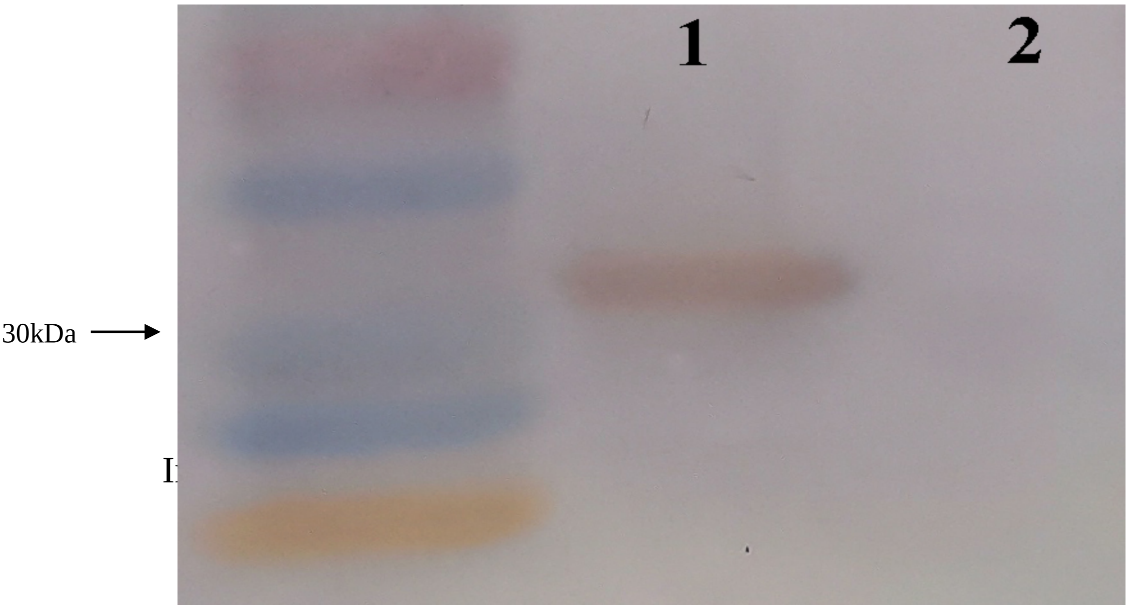

30kDa

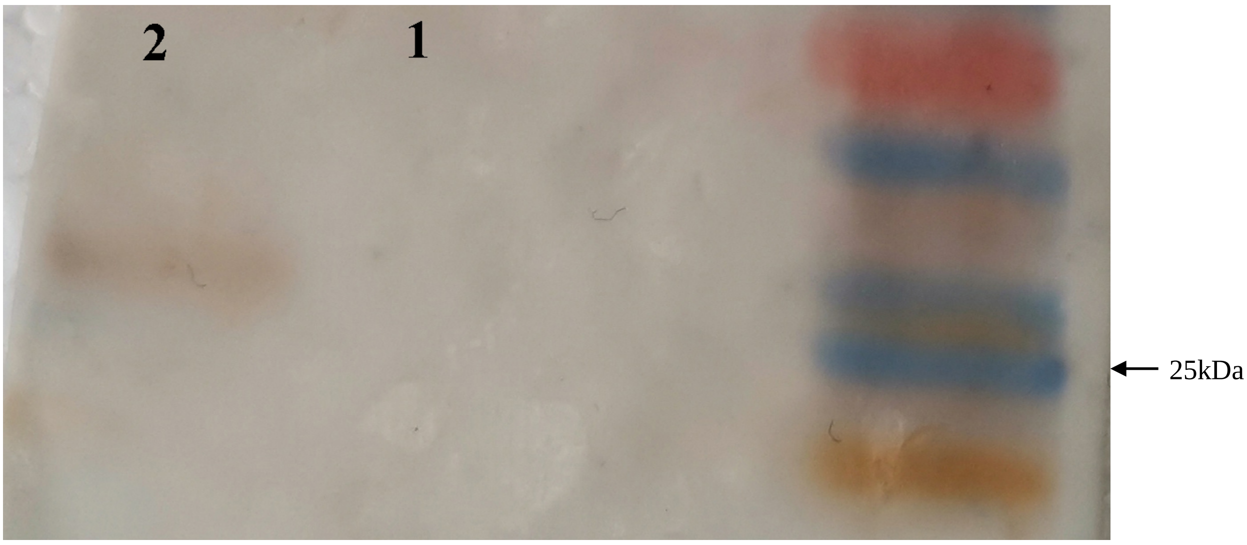

25kDa

1: FLAG:RsVAP and egfp were co-expressed in tobacco leaves; 2: FLAG:RsVAP and HA:LeRabA1d were co-expressed in tobacco leaves. IP: FLAG, tobacco leaf proteins immunoprecipitated by Anti-FLAG antibodies; Input: total tobacco leaf proteins; IB: FLAG, Western blot detection with Anti-FLAG as the primary antibody; IB: GFP, Western blot detection with Anti-GFP as the primary antibody; IB: HA, Western blot detection with Anti-HA as the primary antibody
