# Supplementary figures and images for: A Venom Allergen-Like Protein, RsVAP, the First Discovered Effector Protein of Radopholus similis That Inhibits Plant Defense and Facilitates Parasitism
Source: Int J Mol Sci. 2021 Apr 30;22(9):4782. doi: 10.3390/ijms22094782 (PMC8125365; doi:10.3390/ijms22094782)

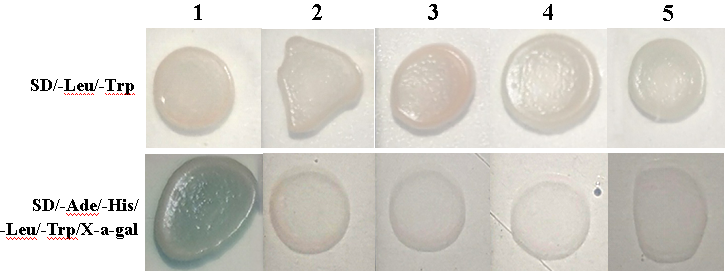

Supplement: Supplementary file 1 [file ijms-22-04782-s001.zip › supplementary material/Figures/Figuer12.png]

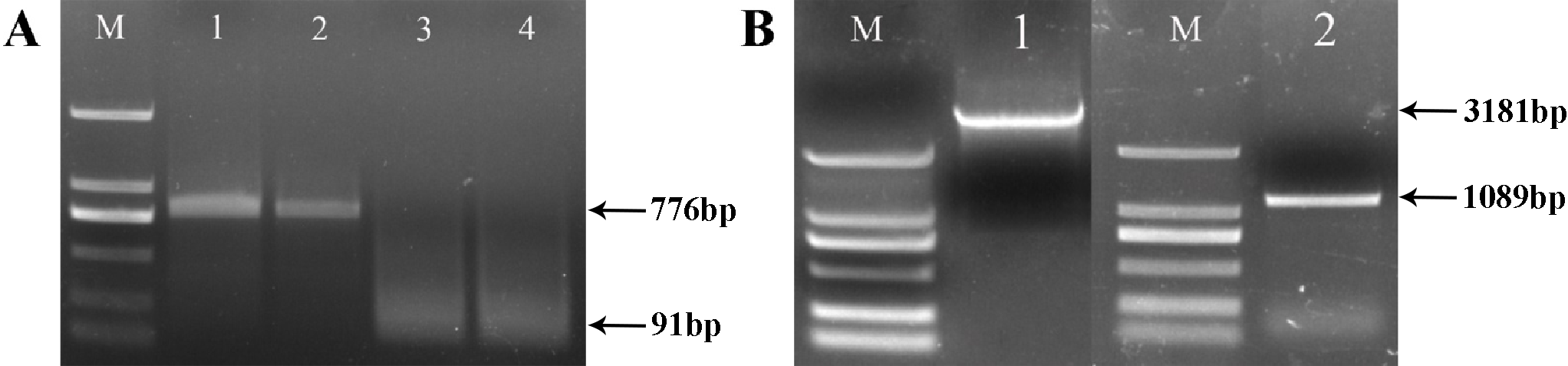

Supplement: Supplementary file 1 [file ijms-22-04782-s001.zip › supplementary material/Figures/Figure1.tif]

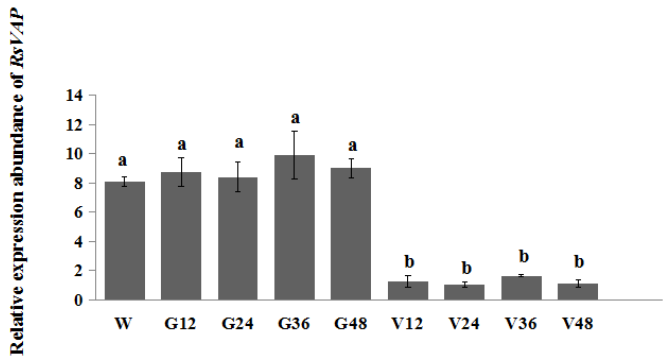

Supplement: Supplementary file 1 [file ijms-22-04782-s001.zip › supplementary material/Figures/Figure10.png]

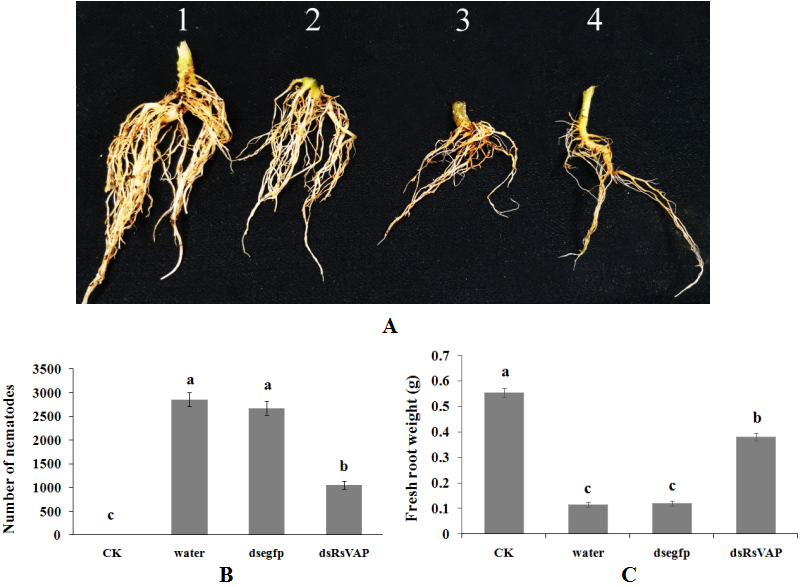

Supplement: Supplementary file 1 [file ijms-22-04782-s001.zip › supplementary material/Figures/Figure11.png]

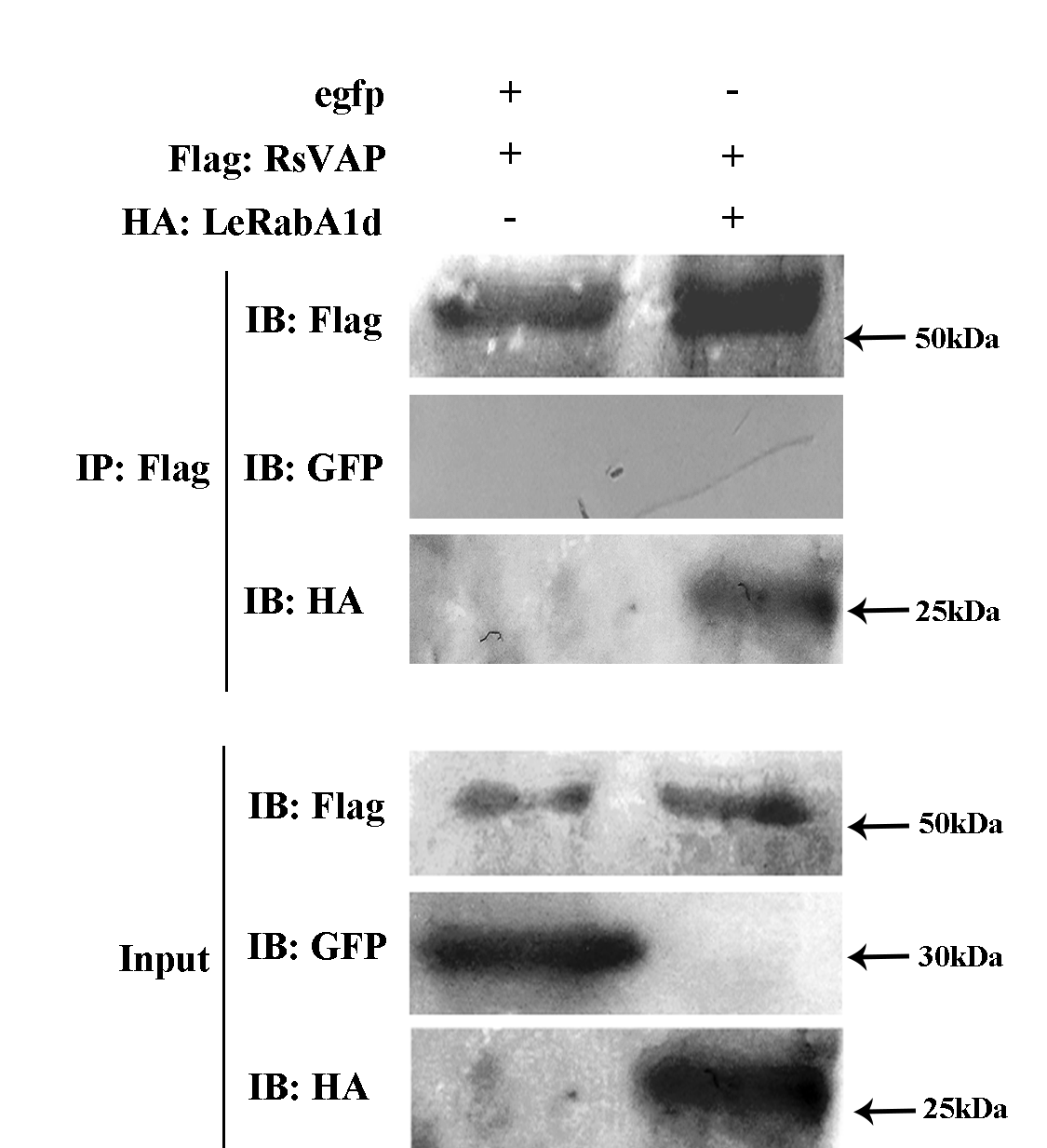

Supplement: Supplementary file 1 [file ijms-22-04782-s001.zip › supplementary material/Figures/Figure13.tif]

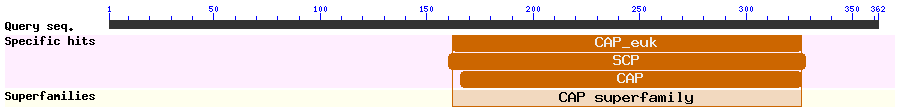

Supplement: Supplementary file 1 [file ijms-22-04782-s001.zip › supplementary material/Figures/Figure2.png]

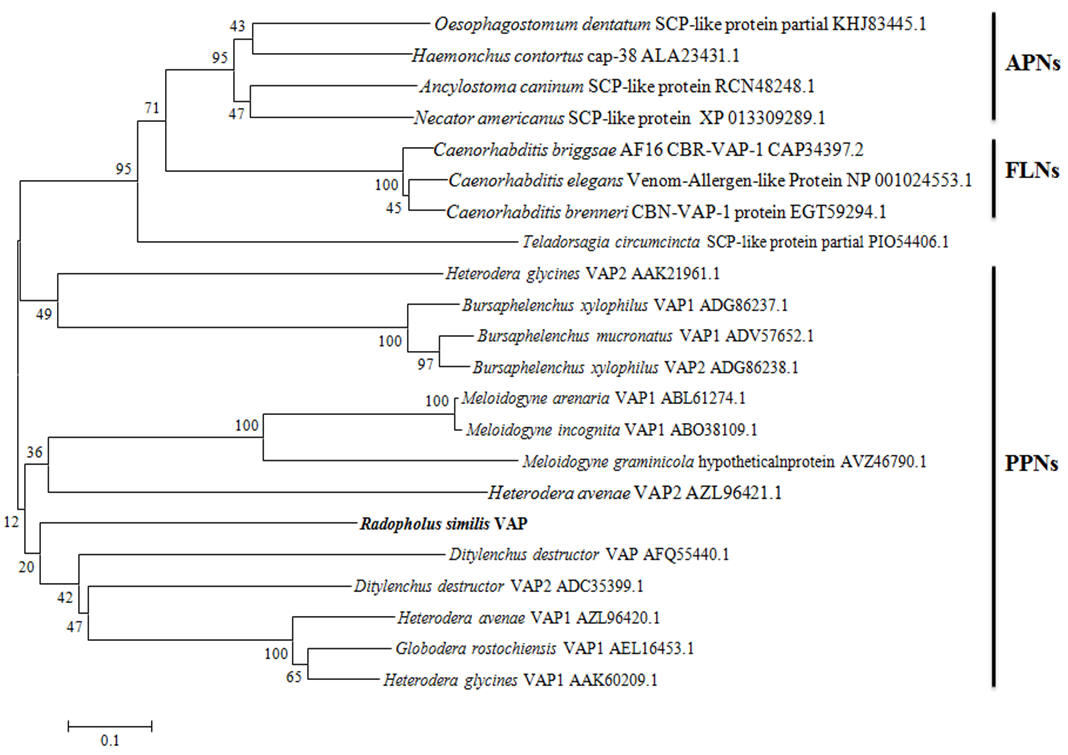

Supplement: Supplementary file 1 [file ijms-22-04782-s001.zip › supplementary material/Figures/Figure3.tif]

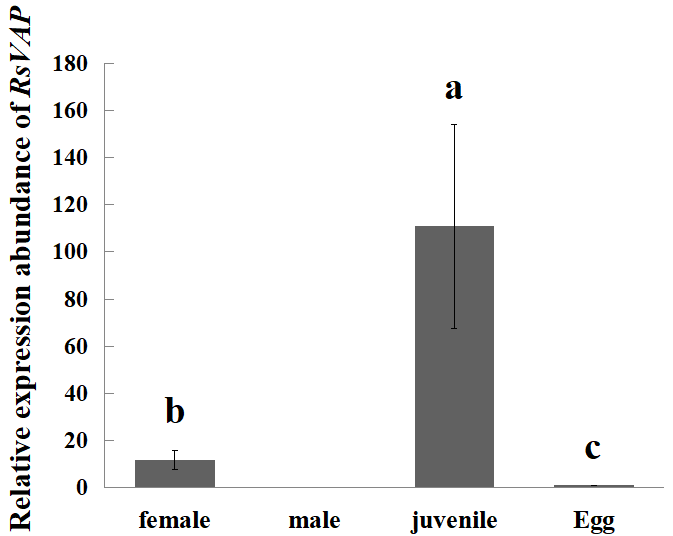

Supplement: Supplementary file 1 [file ijms-22-04782-s001.zip › supplementary material/Figures/Figure4.png]

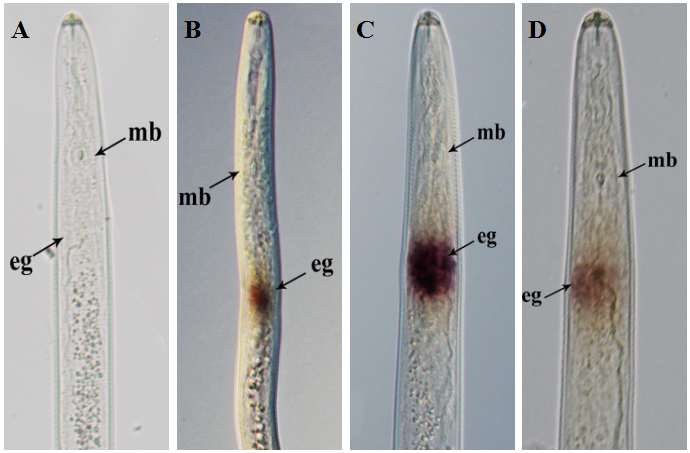

Supplement: Supplementary file 1 [file ijms-22-04782-s001.zip › supplementary material/Figures/Figure5.png]

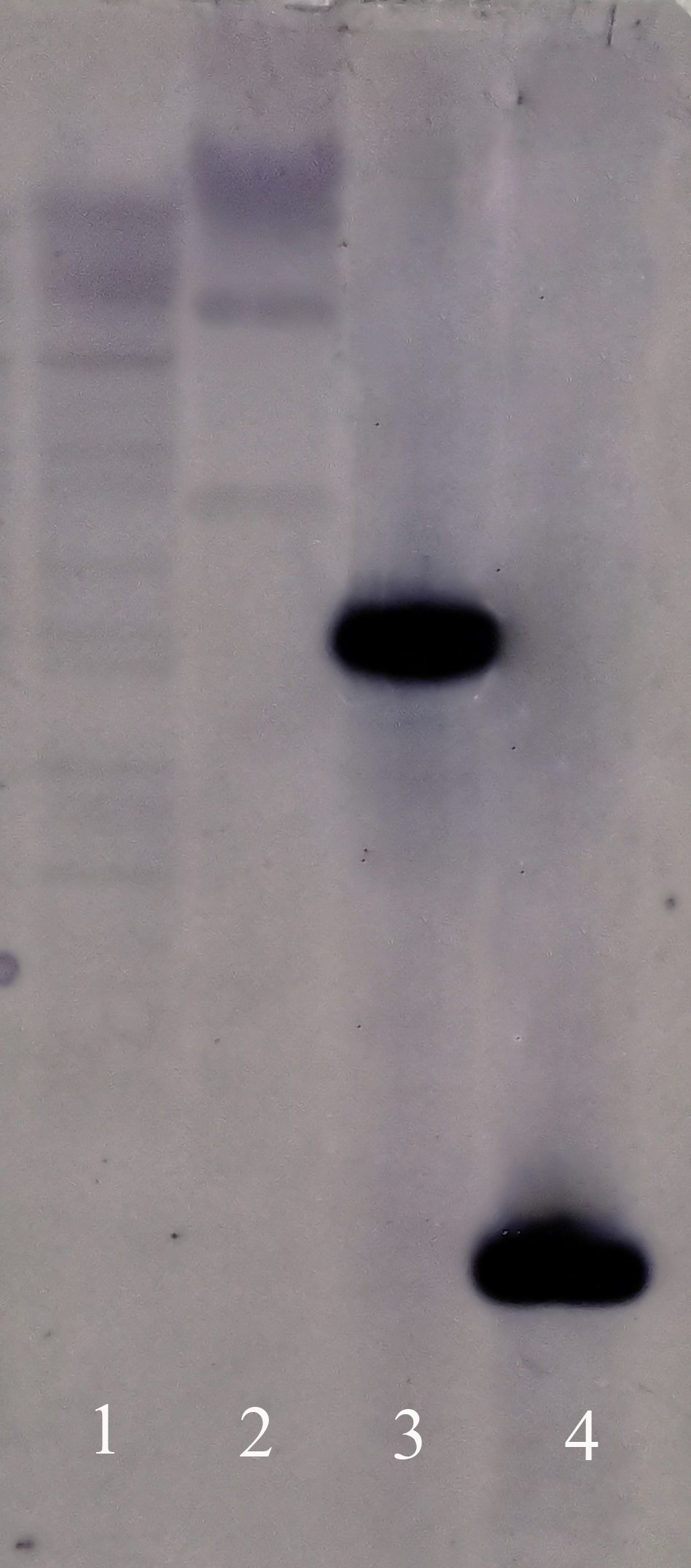

Supplement: Supplementary file 1 [file ijms-22-04782-s001.zip › supplementary material/Figures/Figure6.jpg]

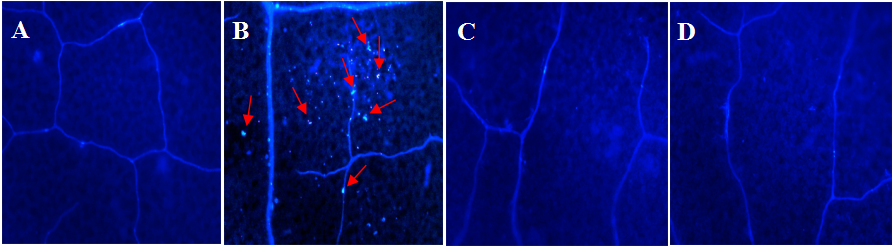

Supplement: Supplementary file 1 [file ijms-22-04782-s001.zip › supplementary material/Figures/Figure7.png]

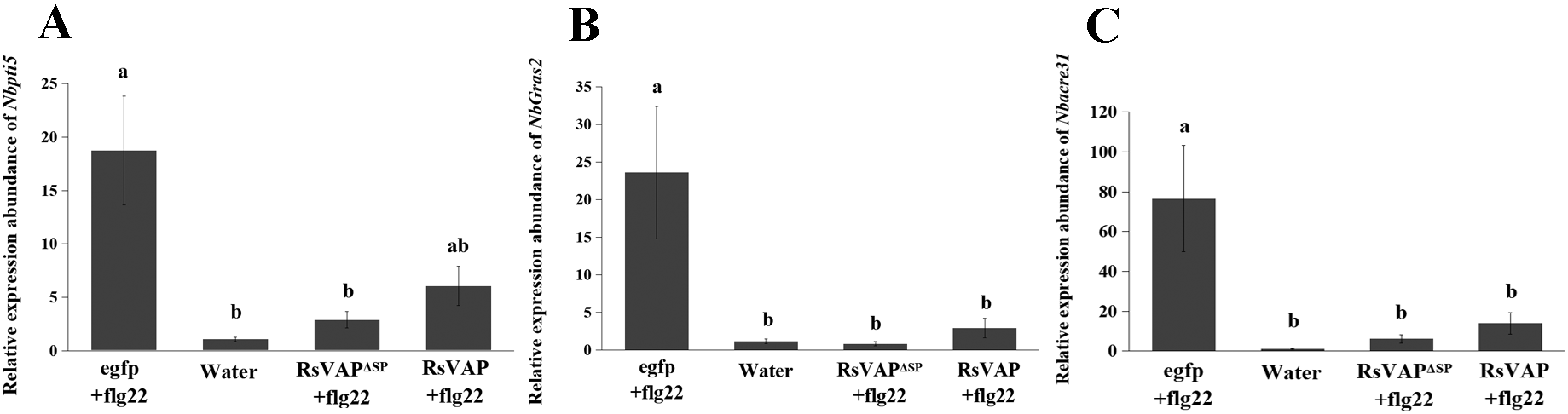

Supplement: Supplementary file 1 [file ijms-22-04782-s001.zip › supplementary material/Figures/Figure8.tif]

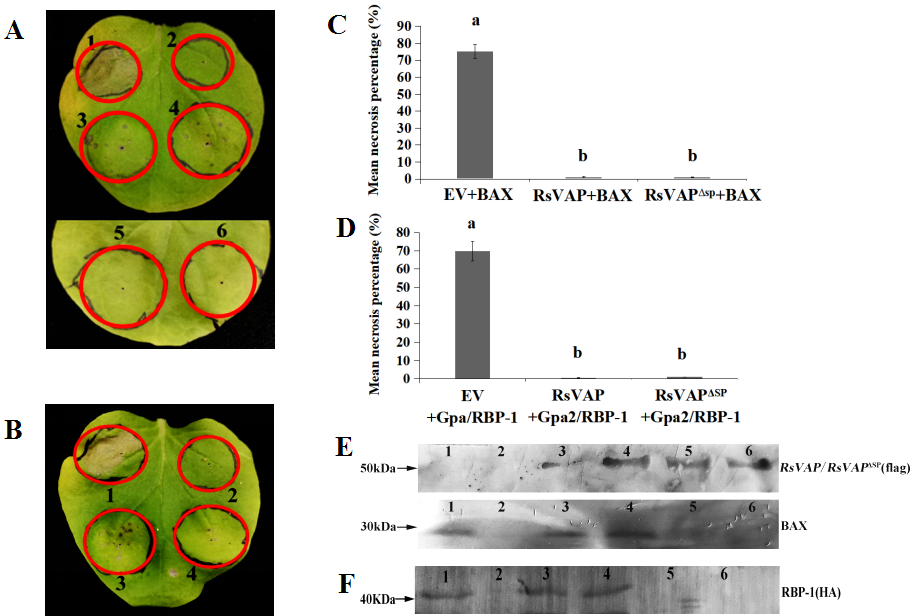

Supplement: Supplementary file 1 [file ijms-22-04782-s001.zip › supplementary material/Figures/Figure9.png]

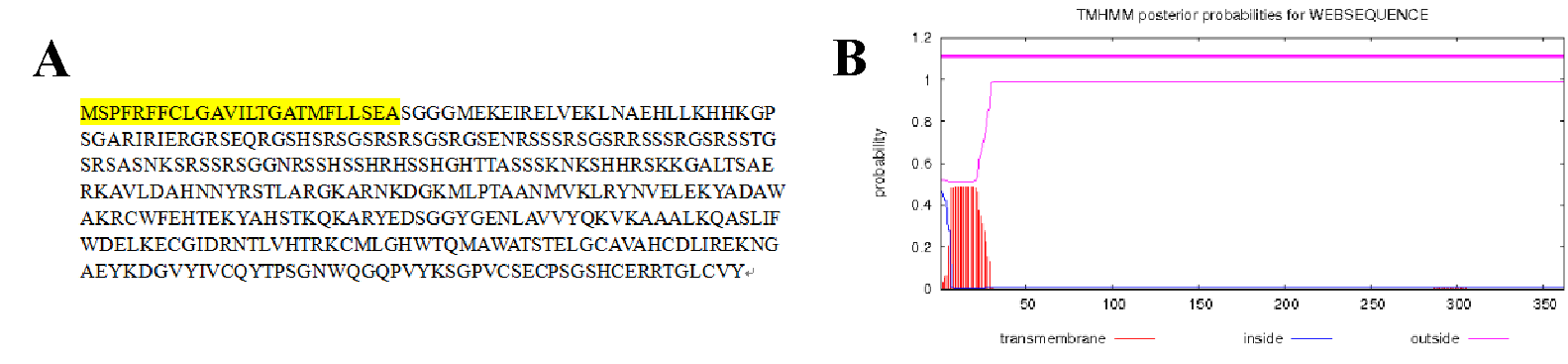

Supplement: Supplementary file 1 [file ijms-22-04782-s001.zip › supplementary material/Figures/FigureS1.tif]

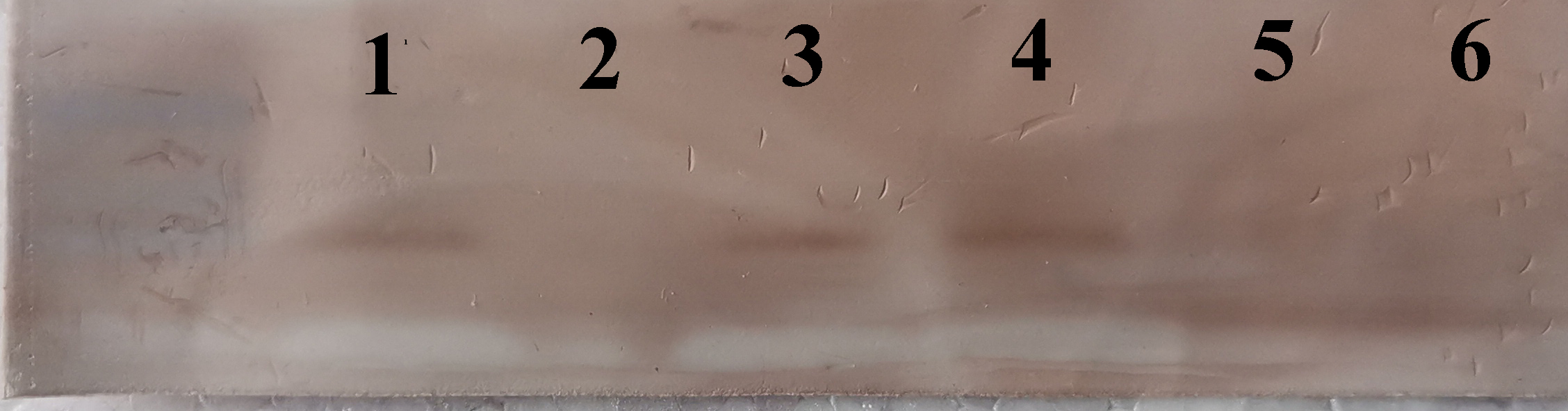

Supplement: Supplementary file 1 [file ijms-22-04782-s001.zip › supplementary material/western-blot/BAXwestern.jpg]

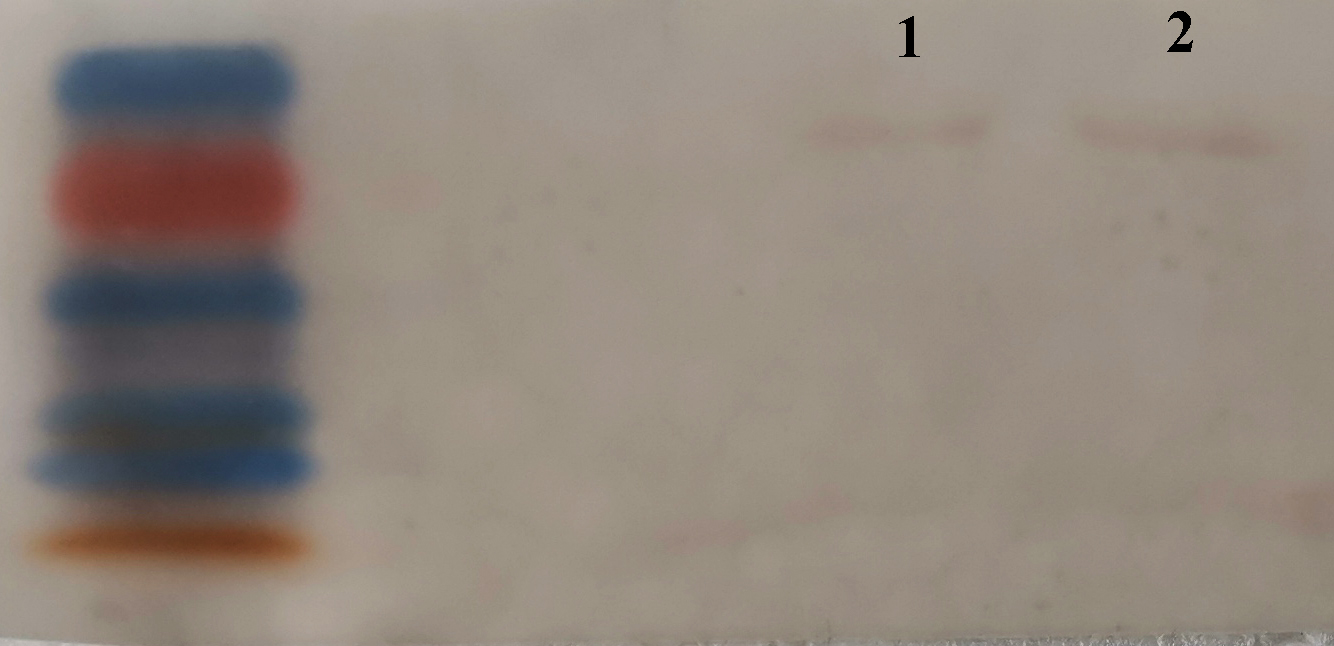

Supplement: Supplementary file 1 [file ijms-22-04782-s001.zip › supplementary material/western-blot/Input-IBflag.jpg]

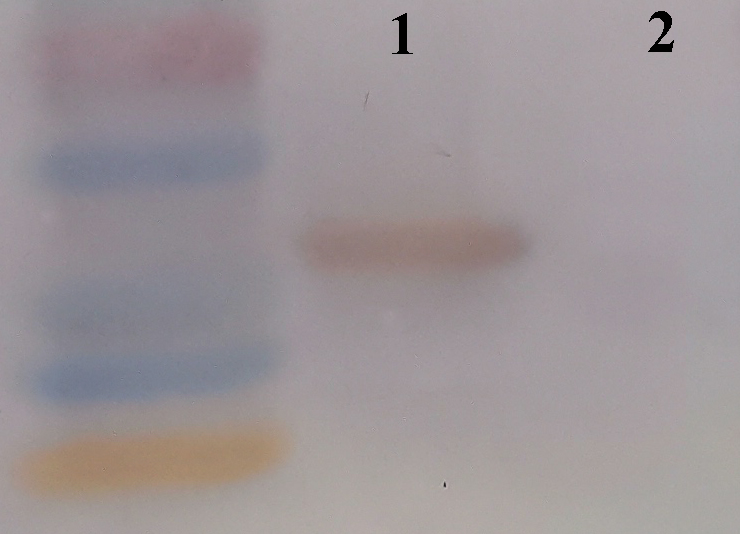

Supplement: Supplementary file 1 [file ijms-22-04782-s001.zip › supplementary material/western-blot/Input-IBGFP.jpg]

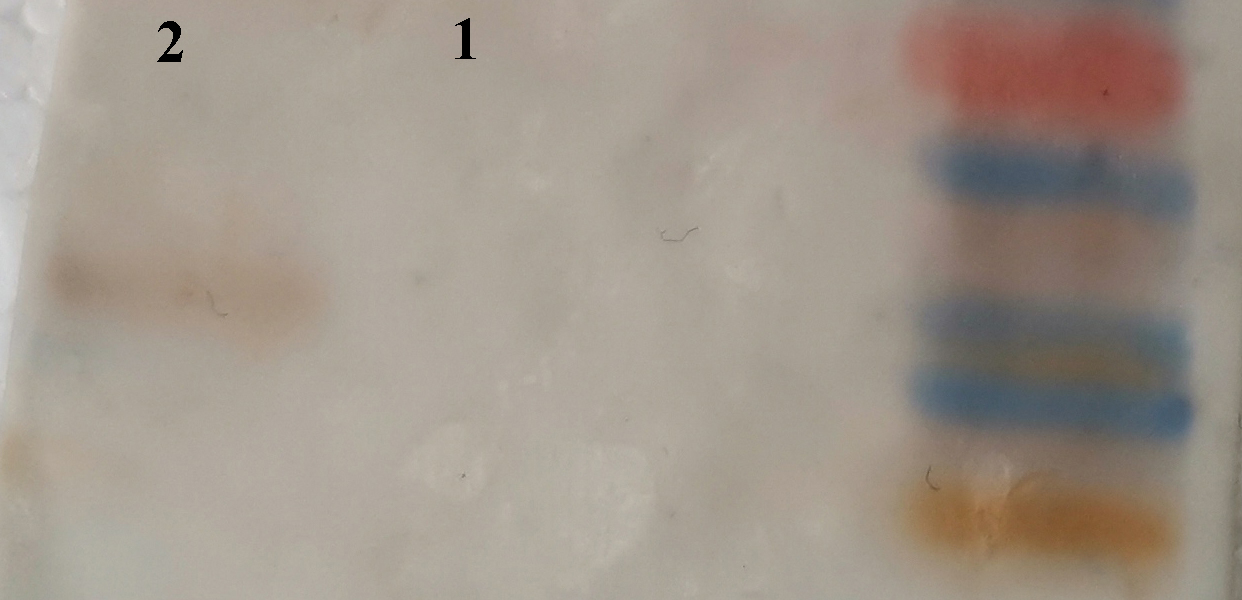

Supplement: Supplementary file 1 [file ijms-22-04782-s001.zip › supplementary material/western-blot/Input-IBHA.jpg]

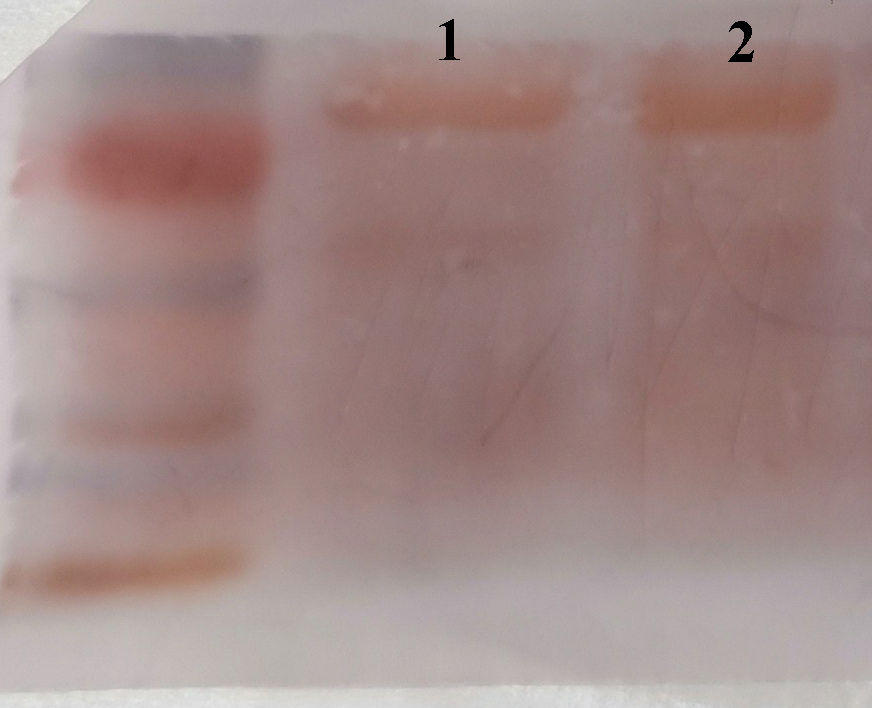

Supplement: Supplementary file 1 [file ijms-22-04782-s001.zip › supplementary material/western-blot/IPflag-IBFlag.jpg]

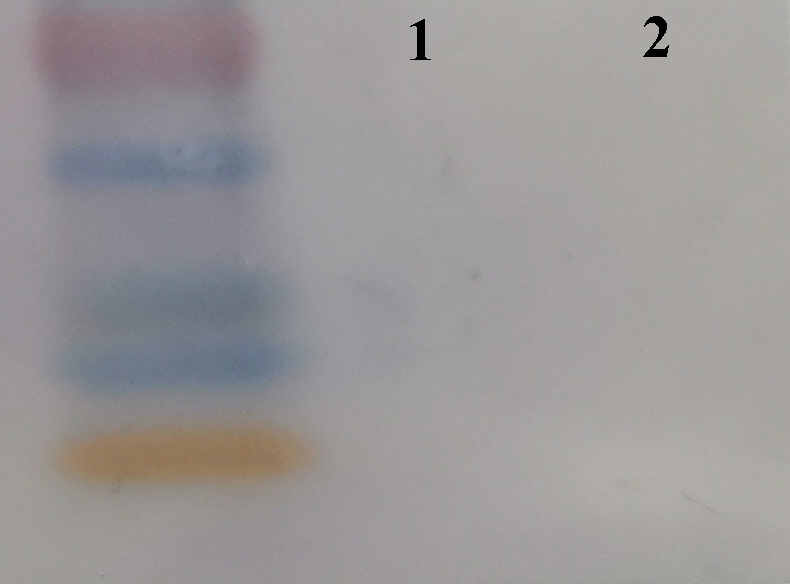

Supplement: Supplementary file 1 [file ijms-22-04782-s001.zip › supplementary material/western-blot/IPflag-IBGFP.jpg]

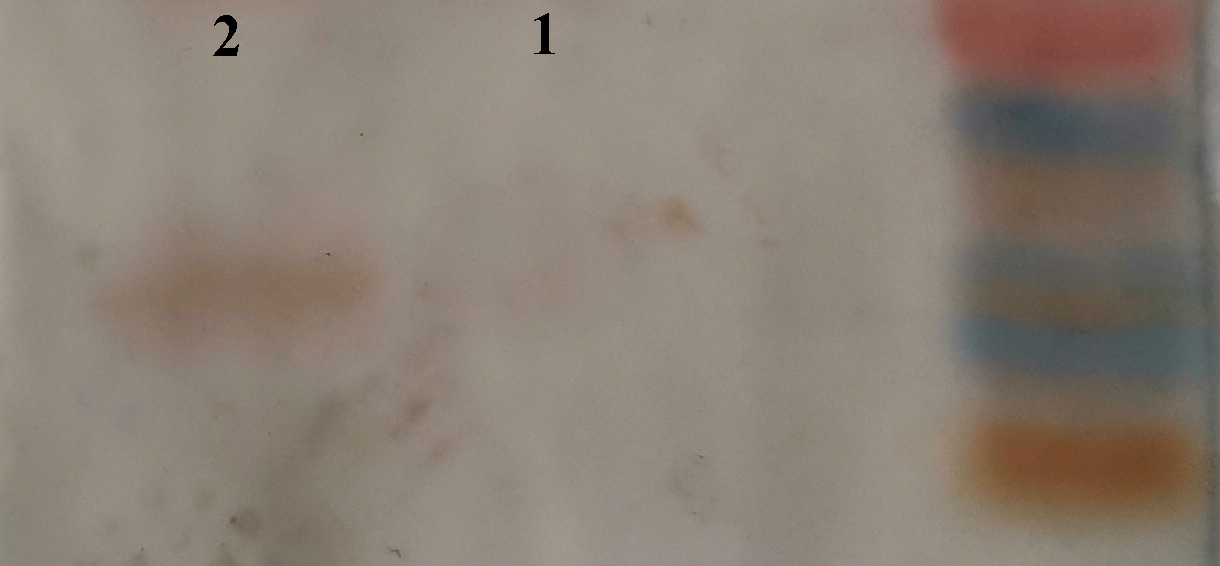

Supplement: Supplementary file 1 [file ijms-22-04782-s001.zip › supplementary material/western-blot/IPflag-IBHA.jpg]

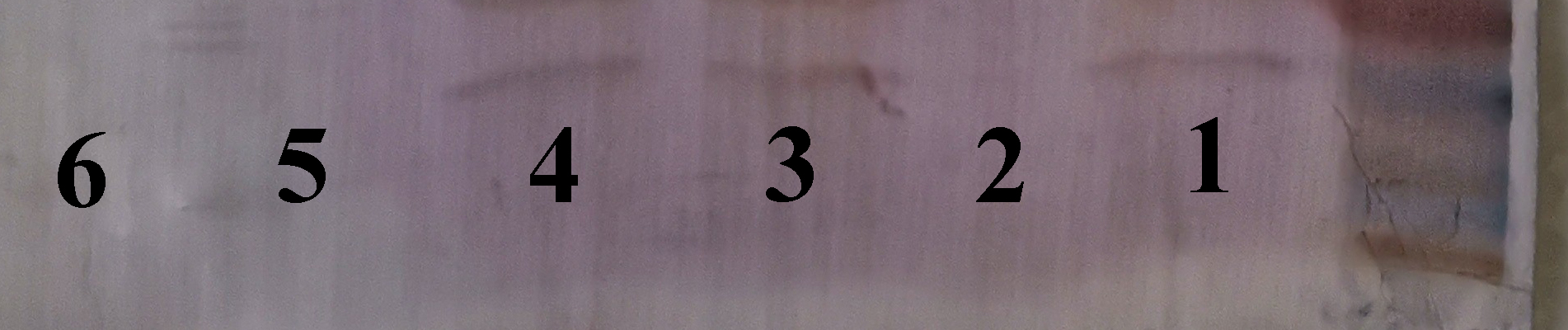

Supplement: Supplementary file 1 [file ijms-22-04782-s001.zip › supplementary material/western-blot/RBP-1western.jpg]

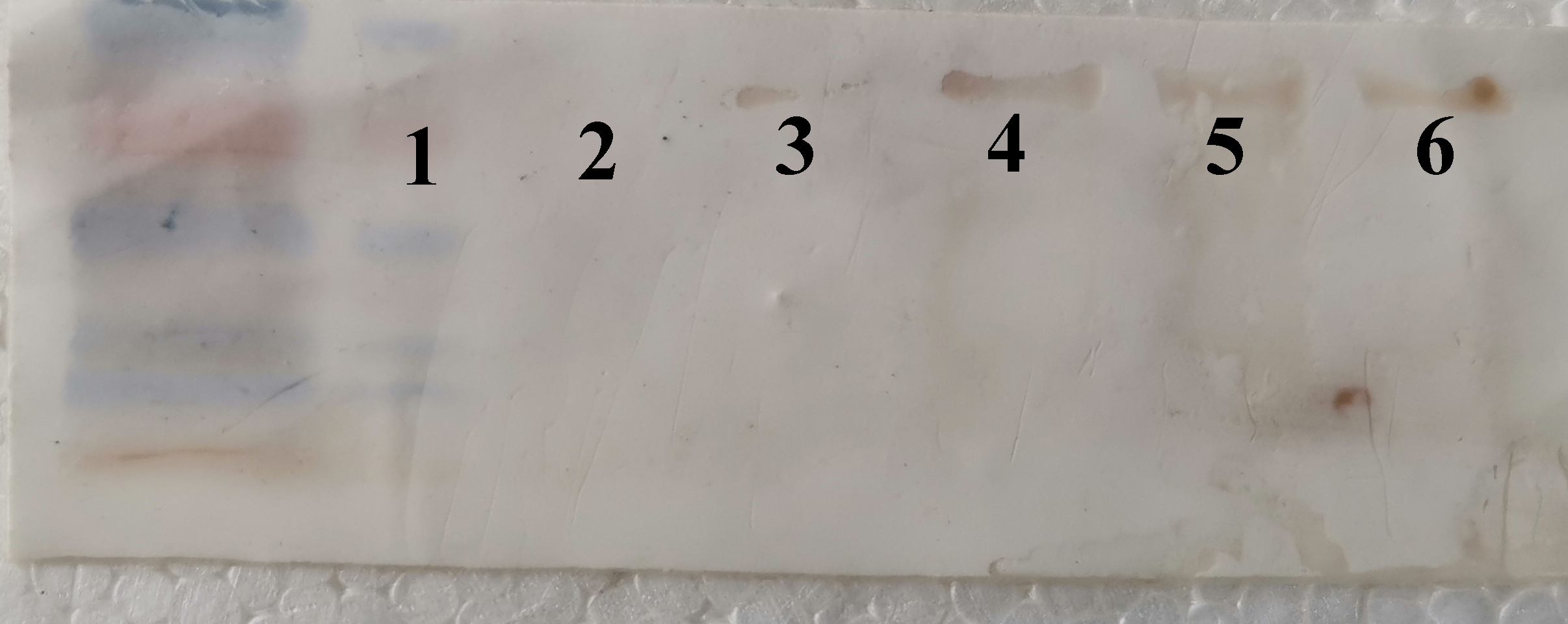

Supplement: Supplementary file 1 [file ijms-22-04782-s001.zip › supplementary material/western-blot/Rsvapwestern.jpg]
